# Type 2 diabetes mellitus in adults: pathogenesis, prevention and therapy

**DOI:** 10.1038/s41392-024-01951-9

**Published:** 2024-10-02

**Authors:** Xi Lu, Qingxing Xie, Xiaohui Pan, Ruining Zhang, Xinyi Zhang, Ge Peng, Yuwei Zhang, Sumin Shen, Nanwei Tong

**Affiliations:** Department of Endocrinology and Metabolism, Research Centre for Diabetes and Metabolism, West China Hospital, Sichuan University, Chengdu, China

**Keywords:** Endocrine system and metabolic diseases, Metabolic disorders, Therapeutics

## Abstract

Type 2 diabetes (T2D) is a disease characterized by heterogeneously progressive loss of islet β cell insulin secretion usually occurring after the presence of insulin resistance (IR) and it is one component of metabolic syndrome (MS), and we named it metabolic dysfunction syndrome (MDS). The pathogenesis of T2D is not fully understood, with IR and β cell dysfunction playing central roles in its pathophysiology. Dyslipidemia, hyperglycemia, along with other metabolic disorders, results in IR and/or islet β cell dysfunction via some shared pathways, such as inflammation, endoplasmic reticulum stress (ERS), oxidative stress, and ectopic lipid deposition. There is currently no cure for T2D, but it can be prevented or in remission by lifestyle intervention and/or some medication. If prevention fails, holistic and personalized management should be taken as soon as possible through timely detection and diagnosis, considering target organ protection, comorbidities, treatment goals, and other factors in reality. T2D is often accompanied by other components of MDS, such as preobesity/obesity, metabolic dysfunction associated steatotic liver disease, dyslipidemia, which usually occurs before it, and they are considered as the upstream diseases of T2D. It is more appropriate to call “diabetic complications” as “MDS-related target organ damage (TOD)”, since their development involves not only hyperglycemia but also other metabolic disorders of MDS, promoting an up-to-date management philosophy. In this review, we aim to summarize the underlying mechanism, screening, diagnosis, prevention, and treatment of T2D, especially regarding the personalized selection of hypoglycemic agents and holistic management based on the concept of “MDS-related TOD”.

## Introduction

Diabetes is a heterogeneous syndrome characterized by defined hyperglycemia which is classified as type 1 diabetes (T1D), type 2 diabetes (T2D), specific types of diabetes and gestational diabetes mellitus.^1^ T2D is a disease characterized by a nonautoimmune heterogeneously progressive loss of adequate islet β cell insulin secretion frequently in the presence of insulin resistance (IR) and metabolic syndrome (MS). We think that “metabolic” or “metabolism” is a description of physiological phenomena which are not able to indicate normal or abnormal phenomena, so we adopt the proper term “metabolic dysfunction syndrome (MDS)” to replace MS. T2D accounts for 96% of diabetes and is one of the important noncommunicable chronic diseases that seriously threaten human health, without totally clear cognition on pathogenesis.

The continuous hyperglycemia can induce target organ damage (TOD) by increasing the risk of panvascular disease, including microvascular disease (such as diabetic retinopathy, nephropathy and neuropathy), and atherosclerotic macrovascular disease (cardiovascular, cerebrovascular, and other peripheral vascular diseases). Unlike classical T1D with almost only hyperglycemia, T2D is just one component of MDS, and is often accompanied by other components of MDS, such as overweight/obesity (preobesity might be a more appropriate term instead of “overweight” since obesity is not determined only by weight), metabolic dysfunction associated steatotic liver disease (MASLD), dyslipidemia. They are usually considered as the upstream diseases of T2D. Among them, preobesity/obesity, MASLD and some coexisting hypertension, closely related to poor lifestyle, are independent risk factors for the development of cardiovascular-kidney-metabolic syndrome and T2D,^2^ indicating their preventable characteristic.

The realization of T2D as downstream disease of MDS suggests that “metabolic complications or “MDS-related TOD” may be more reasonable instead of so-called “chronic diabetic complications”. This updated philosophy is crucial for the management of T2D, emphasizing that TOD is not only caused by hyperglycemia. The used term “chronic diabetic complications” is misleading for both doctors and patients, leading to the blood glucose-focused management, while ignoring the development of TOD related to other metabolic disorders in the MDS. Based on this concept, the treatment should be holistic management to protect the target organs of MDS.

This review provides an overview of the epidemiology, diagnosis, screening, and prevention of T2D, with an emphasis on T2D pathophysiology and molecular mechanisms, as well as holistic management for T2D based on the concept of “MDS-related TOD”.

## Epidemiology

Diabetes has become a worldwide health burden due to its high incidence, disability and mortality, which is estimated to be the eighth leading cause of death combined disability.^3,4^ It is estimated that the annual global health care spending on diabetes among people aged 20–79 was US$ 966 billion in 2021, and is estimated to reach more than $1054 billion by 2045.^5^ In 2021, there were 529 million people of all ages in worldwide living with diabetes, and the global age-standardized prevalence is 6.1%, which increased by 90.5% from 3.2% in 1990, and is expected to reach 9.8%, affecting 13.1 billion people. It is noteworthy that T2D accounts for more than 96% of all.^3^ T2D is a complex multifactorial polygenetic disease that can attribute to many risk factors. Preobesity/obesity are major risk factors for T2D. In 2021, high BMI contribute more than 50% of global T2D disability-adjusted life year (DALY).^3^ Mounting evidence show association between T2D and gender difference.^6^ The global age-standardized diabetes prevalence was higher in males than in females in 2021, with a male-to-female ratio of 1.14 despite it varied from regions.^3^ Age is also an important risk factor for T2D. The global diabetes prevalence peaked in group aged 75–79 years, at 24.4% while less than 1% in group age under 20 years.^3^ Other factors such as dietary risks, environmental or occupational risks, tobacco use, low physical activity alcohol use all account for part of the risk of T2D.^3^ In addition, genetics also play a modest but true role in T2D risk. Studies showed that the estimates of the heritability of T2D range from 30% to 70%, depending on the age of diabetes onset and the glycemic status of cases.^7^ For now, more than 100 T2D-susceptibility genes have been mapped in human including TCF7L2, GCK, KCNJ11, PPARG.^7^

MDS-related TOD including macrovascular diseases and microvascular complications, such as chronic kidney disease (CKD), retinopathy, neuropathy, along with diabetic foot ulcerations (DFU), are responsible for much of the burden associated with diabetes.^8^ CKD, which has been recognized as primary factors contributing to elevated mortality and DALYs among diabetic patients,^9^ occurs in 20–40% diabetic patients and it’s still rising. It is estimated approximately 50% and 34% of patients with diabetes will be affected by diabetic neuropathy (DN) and DFU over the course of their lifetime, respectively.^10,11^ About 20% of people with a DFU will undergo a lower extremity amputation and the 5-year mortality rate for them is greater than 70%.^10^ Diabetic retinopathy (DR), occurred in 22% of diabetes patients and 6% suffer from vision threatening DR which might lead to irreversible vision impairment even blindness.^12^

## Pathophysiology and signaling pathways involved

**Fig. 1:**
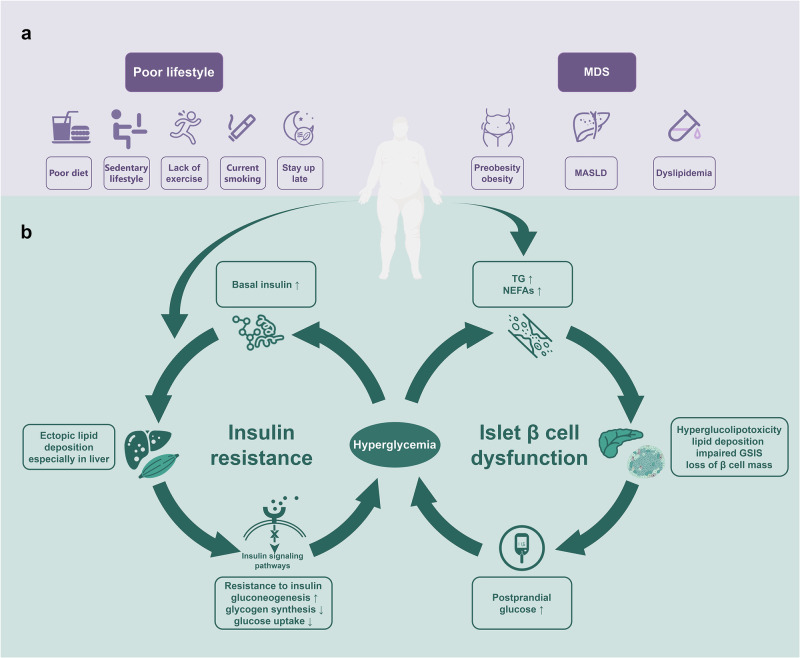
Vicious cycle of hyperglycemia. Poor lifestyle and/or metabolic dysfunction syndrome leads to elevated triglycerides, non-esterified fatty acids (**a**). Excessive lipids is deposited in non-adipose tissue, blocking the insulin signaling pathways, then resulting in insulin resistance, especially in the liver which increase the liver’s glucose production and weakens uptake of glucose, thereby increasing blood glucose and basal insulin levels, and the elevated insulin promotes lipid deposition, further aggravating insulin resistance and forming a vicious circle; elevated glucose and lipids produce hyperglucolipotoxicity to islet β cells and lipid deposition in islets, damaging the secretion function and number of pancreatic β cells, and further increasing blood glucose (**b**). MDS metabolic dysfunction syndrome, MASLD metabolic associated steatotic liver disease, TG triglycerides, NEFAs non-esterified fatty acids, GSIS glucose-stimulated insulin secretion

T2D, usually accompanied by other manifestations of MDS, is a complex metabolic disease with multiple underlying mechanisms not fully understood, while IR and β cell dysfunction are two core pathophysiological mechanisms^13,14^ (Fig. 1). Inflammation, ectopic lipid deposition, endoplasmic reticulum stress (ERS), and oxidative stress are involved in the onset and progression of T2D and TOD by impairing insulin sensitivity and/or β cell dysfunction, reciprocal with metabolic disorders.

### IR and insulin signaling pathways

IR is defined as the loss of the ability of target tissues to respond to insulin signals, resulting in hyperinsulinemia and associated with many metabolic disorders in MDS, such as obesity, MASLD and hypertension. It often presents before T2D occurs and is thus considered as a risk factor.

The occurrence of IR varies by sex and ethnicity. Women demonstrate lower prevalence of T2D and prediabetes in younger ages as well as decreased mortality due to diabetes compared to males despite higher BMI in women.^15^ The incidence of IR in Asian-Indians is approximately 2 to 3 times greater than that in other races,^16^ possibly related to their phenotypes of high fat mass and low lean mass, indicating higher insulin levels.^17^

Some genetic factors are also found to be involved. For example, individuals with defects in genes mediating glucose transporter (GLUT)4 translocation have a genetic susceptibility to IR for the aberrant glucose uptake,^18^ while GLUT4 expression in adipocyte is reduced in T2D and prediabetes.^19^ Gene expression differences involved in O-GlcNAcylation and hexosamine biosynthesis pathway have also been detected between diabetic and nondiabetic individuals,^20^ probably because multiple molecules in insulin signaling pathways can be modified by glycosylation which in turn reduces phosphorylation, leading to inhibition of insulin signaling.^20,21^ Pregestational diabetes exposes oocytes to hyperglycemia. It downregulates ten-eleven-translocation protein 3 (TET3) and impairs the DNA demethylation at the paternal alleles of several insulin secretion genes such as glucokinase (GCK), thus making the offspring susceptible to impaired glucose tolerance and diabetes.^22^

#### Insulin signaling pathways

**Fig. 2:**
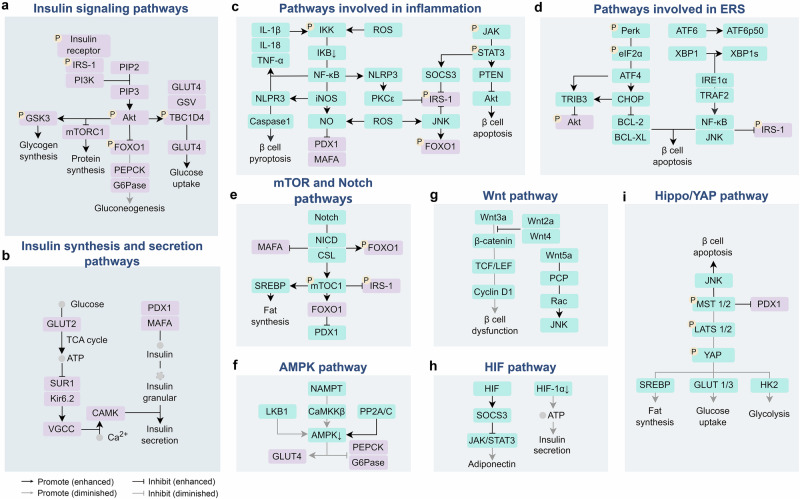
Signaling pathways involved in T2D. In T2D, insulin signaling pathways (**a**) and insulin synthesis and secretion pathways (**b**) are affected by inflammatory pathways (**c**), ERS (**d**), and other pathways such as mTOR and notch pathways (**e**), AMPK pathway (**f**), Wnt pathway (**g**), HIF pathway (**h**), Hippo/YAP pathway (**i**)

Physiologically, insulin binds to the extracellular α subunit of insulin receptor, a receptor tyrosine kinase which recruits and phosphorates insulin receptor substrate (IRS)-1, thus activating phosphoinositide 3-kinase (PI3K)/protein kinase B (Akt) signaling and diverse downstream pathways to maintain the glucose homeostasis.^23^ Firstly, activated Akt induces the translocation of GLUT4 vesicle from cytoplasm to membrane, promoting glucose uptake.^24^ Secondly, activated Akt phosphorylates forkhead box O1 (FOXO1), a transcription factor upregulating gluconeogenesis rate-limiting enzyme such as phosphoenolpyruvate carboxykinase (PEPCK) and glucose-6-phosphatase (G6Pase), and translocate it from nucleus to cytoplasm, which inactivate its transcriptional activity, thus inhibiting gluconeogenesis.^25^ Thirdly, activated Akt directly phosphorylates and inactivates glycogen synthase kinase 3 (GSK3), thus promoting glycogen synthesis. In addition, activated Akt can relinquish the inhibition of mammalian target of rapamycin (mTOR) signaling pathways and promote proteins synthesis (Fig. 2a).

#### Overview of IR

The metabolites of chronic overnutrition, such as high glucose and non-esterified fatty acids (NEFAs), interfere with the activation of insulin receptor and its subsequent IRS-1/PI3K/Akt2 pathways, leading to the development of chronic inflammation in adipose tissue and ectopic lipid deposition in the liver and muscles, along with ERS and oxidative stress, etc. These alterations in target organs interact with each other to aggravate metabolic disorders, ultimately leading to IR. Many of the pathophysiology and the signaling pathways involved in this process are also shared by the development of MDS-related TOD.

In the early stage of IR, β cells work overload to increase insulin secretion to maintain blood glucose stability, with the compensatory capacity more genetically determined.^26^ As the disease progresses, β cells are no longer able to secrete enough insulin to compensate for the abnormally high blood glucose, leading to progression to prediabetes or T2D.^27^

#### Inflammation

Obesity associated with overnutrition induces macrophage infiltration and chronic hypoxia, causing low-grade inflammation in the adipose tissue^28^ and the release of proinflammatory factors such as interleukin (IL)-1β and tumor necrosis factor α (TNF-α),^29,30^ thus impairing insulin signaling pathway in a number of ways.

Firstly, they downregulate the level of insulin signaling molecules at the transcriptional level. Secondly, they activate multiple signaling pathways associated with inflammation (e.g. NF-κB, JAK/STAT and JNK), leading to impaired activation of the molecules. Thirdly, they also accelerate ceramide synthesis, aggravating ectopic lipid deposition.^31,32^

In diabetic peripheral neuropathy (DPN), macrophages infiltrate to initiate repairment on nerve injury, with more cytokine and chemokine released.^33^ This highly inflammatory environment intensifies existing oxidative stress, further aggravating nerve damage.^34^

##### NF-κB signaling pathway

Nuclear factor-kappa B (NF-κB) transcription factors are critical regulators of immunity, stress responses, apoptosis and differentiation.^35^ In T2D, the NF-κB pathway can be activated directly by excess metabolites and multiple inflammatory factors.^36^ Those stimuli phosphorylate IκB kinase (IKK) complex,^37^ and induce degradation of IκB, thus releasing NF-κB to translocate to nucleus. NF-κB then promotes the expression of inducible nitric oxide synthase (iNOS) and the production of nitric oxide (NO), and finally inhibits the activation of IRS-1. It also upregulates the transcription of IL-1β, IL-18, and TNF-α, forming positive feedback.

Besides, activation of IKKβ, a component of IKK complex, can also directly phosphorylate IRS-1 serine to interfere its activation. What’s more, the NOD-like receptor family pyrin domain-containing 3 (NLRP3), also upregulated by the activation of NF-κB,^38^ forms inflammasome and activates protein kinase C epsilon (PKCε),^39^ thus imparting IRS-1^40^ (Fig. 2c).

NF-κB activation also participated in the MDS-related TOD. Firstly, activated NF-κB and NLRP3 inflammasome in DN promotes inflammation and increases fibronectin and collagen deposition in kidney,^41,42^ leading to thickening glomerular basement membrane, glomerular sclerosis, podocyte damage, and ultimately kidney fibrosis.^43^ Secondly, Activation of NF-κB also leads to wound inflammation and promotes apoptosis of human endothelial cells by enhancing caspase-3 activity, thus delaying wound healing in DFU.^44^ Thirdly, retinal NF-κB in diabetes is activated at the beginning of retinopathy, initiating proapoptotic procedures,^45^ and stay energetic while interacts with reactive oxygen species (ROS) even if the apoptotic process of retinal capillary cells speeds up.^46,47^ Elevated NLRP3 and IL-1β in the retina of proliferative diabetic retinopathy (PDR) patients are involved in the formation of late pathological neointima and lead to apoptosis of pericytes in the retina.^48^

##### JAK/STAT signaling pathway

The janus kinase (JAK)/signal transducers and activators of transcription (STAT) signaling pathway is involved in cell proliferation, differentiation, apoptosis, and inflammation response. It serves as an important downstream mediator for various cytokines, hormones, and growth factors. When they bind to their receptors, JAK proteins are recruited intracellularly, dimerized and activated through autophosphorylation. These activated JAKs then phosphorylate STAT proteins, which then translocate to the nucleus where they regulate a set of gene transcription.^49^

Elevated inflammatory factors in T2D such as IL-6 and interferon-γ (IFN-γ) cand bind to their receptors, activate JAK2/STAT3, subsequently upregulates the expression of suppressor of cytokine signaling 3 (SOCS3), which inhibit IRS-1 cascade.^50,51^ JAK/STAT3 is also found to promote NF-κB cascade^50^ (Fig. 2c).

JAK/STAT3 activation also relates to the MDS-related TOD. During DN, the increased expression of JAK in glomerular podocytes aggravates the persistent inflammatory response of the kidney by activating the STAT3/NF-κB pathway.^52,53^ In DFU, the increasing expression of IL-6 results in a sharp increase in phosphorylated STAT3 levels,^54^ which mediates impaired immune cell activation, recruitment, and survival, resulting in delayed wound healing.^55^

##### JNK signaling pathway

JNK, a subgroup of the mitogen-activated protein kinase (MAPK) family, can be activated by the inflammatory factors, metabolites and the crosstalk between other signaling pathways.^25,56,57^

Activated JNK not only phosphorylates IRS-1 serine to block its proper activation, but also causes phosphorylation and translocation of FOXO1 directly (Fig. 2c).

#### ERS

ERS in insulin target organs has been considered as a major pathogenesis of IR. ERS activating unfolded protein reaction (UPR) pathways is recognized by 3 sensors in endoplasmic reticulum (ER) membrane: protein kinase RNA-like endoplasmic reticulum kinase (PERK), inositol-requiring protein 1α (IRE1α), and cyclic AMP-dependent transcription factor 6α (ATF6α). They promote downstream signaling for alleviating cell survival, or induced apoptosis pathway under sustained UPR.

PERK phosphorylates the eukaryotic translation initiator factor 2 (eIF2), which increase the translation of transcription factor-4 (ATF4) and subsequently upregulating the expression of C/EBP homologous protein (CHOP) and tribbles homolog 3 (TRIB3).^37^ Among them, CHOP inhibits the expression of anti-apoptosis proteins B-cell lymphoma-2 (BCL-2) and B-cell lymphoma-extra-large (BCL-XL), inducing cell apoptosis, while TRIB3 inhibits Akt activity, promoting IR in insulin-target organs. IRE1α binding with tumor necrosis factor receptor-associated factor 2 (TRAF2) activates IKKβ and JNK1, thus promoting chronic inflammation in IR^37,58,59^ (Fig. 2d).

The ERS in the MDS-related TOD promotes the development of the diseases. The X-box-binding protein-1 (XBP1), spliced and activated by IRE1α, is downregulated in glomerular mesangial cells, weakening the inhibitory effect on the phosphatase and tensin homolog (PTEN)/Akt pathway, thereby aggravating kidney damage.^60^ ERS also contributes to DPN and DR, with increased IRE1 in the neurons,^61^ and upregulated CHOP, caspase 12, and phosphorylated JNK in diabetic retina.^62^

#### Oxidative stress

Oxidative stress is a condition that results in the production of ROS or reactive nitrogen species (RNS) that cannot be adequately addressed by normal antioxidant mechanisms.^63^ Prolonged skeletal muscle inactivity promotes mitochondrial dysfunction, resulting in increase in mitochondrial ROS production and leading to protracted oxidative stress and disturbed redox signaling,^64^ which can exacerbate IR through multiple pathways such as JNK and NF-κB pathways (Fig. 2c). However, it does not appear to directly lead to an increase in fasting blood glucose; rather, it converts the carbohydrates into increased liver fat and plasma very-low-density lipoprotein (VLDL) due to hyperinsulinemia.^40,65^

Oxidative stress is also widespread in MDS-related TOD and is broadly associated with other pathophysiological processes. Firstly, excessive accumulation of ROS within mesangial cells under chronic hyperglycemia can lead to cell death by activating mTOR,^66^ sirtuin1,^67^ and XBP1,^60^ resulting in renal dysfunction. Secondly, the ROS attack accelerates apoptosis of retinal tissues and cells and aggravates DR,^68^ with the activated polyol pathway, hexosamine pathway, and PKC pathway, as well as advanced glycation end products (AGEs) accumulation involved in the process.^69^ Thirdly, during the DPN, excess saturated fatty acids (SFAs) and hyperglycemia depolarize mitochondrial membranes and generate ROS,^70^ which eventually produce mitochondrial dysfunction and Schwann cells (SCs) apoptosis.^71^ ROS can also activate downstream effectors like PKCε^72^ and JNK,^73^ which are independently associated with DPN pathology.

#### Ectopic lipid deposition

IR in the liver and muscle is associated with fat mass.^74^ Chronic overnutrition, a sedentary lifestyle and IR in adipose tissue can directly lead to the production and accumulation of excess toxic lipid products (such as diacylglycerol, ceramides, and NEFAs), and the metabolites can aggravate the ectopic lipid deposition through some signaling pathways (such as mTOR and notch). Among them, diacylglycerol activates PKCε and impairs IRS-1 activation, while ceramides activate protein kinase C zeta (PKCζ) and protein phosphatase 2A (PP2A), resulting in dephosphorylation of Akt,^31,32^ and promoting gluconeogenesis.^74,75^

WD40 repeat-containing protein 6 (WDR6) is up-regulated in liver with IR. It phosphorylates upstream stimulatory factor 1 (USF1) and then upregulate fatty acid synthase (FASN), which promotes de novo lipogenesis (DNL) in liver and aggravates hepatic fat deposition.^76^ The altered membrane potential of hepatocytes after adipose deposition causes γ-aminobutyric acid (GABA) release and inhibits the vagus nerve, which is also involved in hyperinsulinemia.^77^ In turn, hyperinsulinemia due to IR in the liver and other target tissues can promote the production of liver fat, resulting in a vicious cycle.

##### mTOR signaling pathway

mTOR, a member of the PI3K-related kinase family, is the core component of two structurally and functionally distinct protein complexes, mTOR complex 1 (mTORC1) and mTOR complex 2 (mTORC2).^78^ mTORC1 participates in protein, glucose and lipid metabolism mainly through phosphorylation and activation of p70S6 kinase 1 (S6K1), while mTORC2, an effector of PI3K, contributes to the activation of Akt.^79^

Chronic exposure to excessive glucose and lipids continuously activates mTOR signaling pathways in liver and muscle, contributing to the development of IR. Firstly, mTORC1/S6K1 activates transcription factor sterol-regulatory element binding proteins (SREBP), promoting fat synthesis. Meanwhile, mTORC1/S6K1 can also phosphorylate IRS-1 serine, thus block its activation^80^ (Fig. 2e).

mTOR pathways are also associated with TOD. Firstly, activated mTOR reduces the antioxidant activity of mesangial cells in the glomerulus, promoting the production of ROS.^66^ It also inhibits the activation of nuclear factor erythroid 2-related factor 2 (NRF2), exacerbating oxidative stress injury to the kidneys.^81^ Secondly, IR blunted mTORC1 signaling in SCs.^70^ It impairs lactate, as a source of energy, shuttling to axons via monocarboxylate transporters,^82^ thus contributing to the nerve damage. Thirdly, activated mTOR enhances wound healing in DFU by upregulating the expression of multiple growth factors,^83^ regulating the accumulation of lipids in endothelial cells and fibroblasts,^84^ inducing epithelial-mesenchymal transition,^85^ and promoting the proliferation and differentiation of fibroblasts.^86^

##### Notch signaling pathway

Notch signaling, intimately involved in embryonic development and maintenance of multicellular organisms in adults, is activated by ligand-receptor interaction between adjacent cells, which leads to successive proteolytic cleavages and releases the notch intracellular domain (NICD). NICD binds to intracellular effector molecules (CSL) to form NICD/CSL transcription complex, activating the notch target genes.^87^

Abnormality of notch signaling is strongly associated with metabolic disorders. Increased notch activity in T2D induces expression of SREBP1 in liver through constitutive activation of mTORC1, promoting fat deposition, and increases expression of G6Pase and PEPCK in liver in a FOXO1-dependent manner, promoting glucogenesis, and ultimately exacerbating IR.^50,88,89^ Inhibition of notch signaling can promote glucose uptake in muscle thus improving glucose tolerance^90^ (Fig. 2e).

In DFU, notch pathway dysfunction can lead to inhibition of cell differentiation, proliferation disorders, and reduced angiogenesis, all of which interfere with diabetic wound healing.^91^

#### Decreased adiponectin and AMPK pathway

Chronic inflammation in adipose tissue leads to a decrease in adiponectin, an insulin-sensitized adipokine released by adipose tissues, which perform physical function through the adenosine 5’-monophosphate-activated protein kinase (AMPK) signaling pathway.^92^ Phosphorylation and activation of AMPK is in response to nutrients and AMP/ATP ratio through the upstream kinases serine/threonine kinase (LKB1) and calcium/calmodulin-dependent protein kinase β (CaMKKβ).^50^ Activated AMPK then promotes glucose uptake through the translocation of GLUT4, suppresses gluconeogenesis by inhibiting G6Pase and PEPCK, and improves lipid metabolism by inhibiting fatty acid synthesis and promoting its oxidation.^93,94^

In T2D, AMPK activity is decreased from several ways. Firstly, less adiponectin from adipose tissue reduces the activation of CaMKKβ due to the downregulation of nicotinamide phosphoribosyl transferase (NAMPT),^74,95^ leading to a weaker AMPK activity, which can be observed long before the onset of diabetes.^96^ Meanwhile, high glucose and inflammation block LKB1 recruitment, activate PP2A/C, and induce degradation of AMPK catalytic subunits through the E3 ubiquitin ligase. AMPK downstream effects are blunted in T2D patients, exacerbating hyperglycemia and hyperlipidemia (Fig. 2f).

AMPK is closely related to mitochondrial autophagy,^97^ which maintains mitochondrial function under stress by timely clearing of damaged mitochondria. Hyperglycemia reduces instantaneous calcium intake, and increases NADPH production, thus inhibiting AMPK.^98^ It leads to the weakening of the ability of renal cells to clear damaged proteins and organelles, thus promoting renal fibrosis in DR.^99^

#### The gut microbiota

Emerging studies have shown that the intestinal microbiota composition of obese people has changed compared with that of lean people, even though the reported changes are inconsistent or even contradictory. An altered composition of the microbiota increases plasma bacterial lipopolysaccharide (LPS) levels, resulting in metabolic endotoxemia.^100^

In addition, a high-fat diet and short-chain fatty acids (the metabolites of the gut microbiota) can alter intestinal permeability, which is a characteristic of human T2D.^100^ Increased permeability exacerbates metabolic endotoxemia, thus triggering immune response and IR through toll-like receptor 4 (TLR4), CD14,^101^ and G protein-coupled receptor (GPR) 41/43.^101^

#### Other signaling pathways with controversial effects

##### Wnt signaling pathway

Wnt signaling pathways is associated with adipogenesis. The canonical Wnt pathway can be activated by the binding of Wnt ligands (secreted glycoproteins) and their receptors, and keep the β-catenin from degradation through altering a range of downstream molecules. Stabilized β-catenin translocates to the nucleus and binds to transcription factors such as T cell factor and lymphoid enhancing factor (TCF/LEF), thus leading to the activation of target genes.^102^ Some non-classical Wnt ligands can activate non-canonical pathways such as Wnt/planar cell polarity (PCP) and Wnt/Ca^+^ pathways, playing different roles.

Wnt signaling pathways are upregulated in IR with controversial effects. Wnt5a activats Wnt/PCP pathway, upregulating Ras-related C3 botulinum toxin substrate 1(Rac), which can activate JNK, thus aggravating IR in adipose tissue.^103^ However, Wnt3a is reported to be capable of activating transcriptional coactivator with PDZ-binding motif (TAZ), which can directly upregulate IRS-1, indicating its potential ability of increasing insulin sensitivity in muscle.^104^ Wnt3a is also found to play a resistance role in the differentiation of preadipocytes into adipocytes^104^ (Fig. 2g).

The Wnt/β-catenin pathway is downregulated in DFU.^105^ It suppresses the biological activity of skin cells and the expression of cytokines, resulting in immune dysfunction of the wound, dysplasia of granulation tissue, and reepithelialization disorders, thus delaying wound healing.^106^

##### HIF signaling pathway

Hypoxia-inducible factors (HIFs), consisting of an oxygen-sensitive α subunit and a constitutively expressed HIF-1β subunit, are major regulators of adaptive responses to hypoxia and directly activate the expression of multiple target genes to maintain cellular oxygen homeostasis.^107^ In the presence of oxygen, HIF-1α is hydroxylated, followed by a ubiquitination reaction, leading to the proteolysis of HIF‐1α through the ubiquitin‐proteasome system; Upon hypoxia, HIF-1α is stabilized and translocates to the nucleus, where it dimerizes with HIF-1β, binds to target genes, and activates gene transcription.^108^

The role of HIF in IR is controversial. In adipose tissue, HIF activation can improve IR by stimulating the thermogenic function of brown adipose tissue (BAT) by regulating mitochondrial biogenesis and glycolysis. However, HIF activates the transcription of SOCS3 which inhibits JAK/STAT3 signaling and thus inhibits the expression of adiponectin (Fig. 2h).

The TRIB3 is induced in DR under ERS, leading to overexpression of HIF1α. In turn, HIF1α regulates GLUT1 expression and, together with TRIB3, increases the influx of glucose in the retina,^109^ aggravating metabolism disorder. In addition, it increases the expression of vascular endothelial growth factor (VEGF) and promotes the formation of retinal blood vessels.^110^

##### Hippo/YAP signaling pathway

Hippo/YAP signaling plays a central role in the cellular proliferation and differentiation. Hippo signaling regulates YAP primarily through large tumor suppressor kinases 1 and 2 (LATS 1/2), which are activated through phosphorylation by mammalian sterile 20-like protein kinases 1/2 (MST1/2). Phosphorylation of YAP results in cytoplasmic localization and degradation, while dephosphorylated YAP proteins translocate to nucleus and induce gene expression through interactions with TEA domain (TEAD) transcription factors.^111^

YAP signaling is involved in glucose and lipid metabolism through crosstalk with other signaling pathway. It promotes glucose uptake by directly upregulating GLUT1/3, promotes glycolysis by upregulating hexokinase 2 (HK2), and promotes lipogenesis by directly interacting with SREBP.^111^ Therefore, abnormal Hippo/YAP pathways may be the potential pathogenesis of IR and other metabolic disease (Fig. 2i).

### β cell dysfunction and pathways involved

Islet β cell dysfunction plays a key role in the progression of T2D, which includes impaired insulin synthesis and secretion, and a reduced mass of β cells. It occurs before the diagnosis of T2D, by when the β cells have been lost approximately 50% compared with that of nondiabetic individuals.^112^

Islet β cells shift from a compensatory increase to reduced function and decreased β cell mass.^27^ In the early stage of T2D, impaired insulin synthesis and secretion plays a major role, with the stimuli reducing glucose-stimulated insulin secretion (GSIS), the basic function of β cells.^74^ Progressively, chronic exposure to high glucose and lipids affects the survival of islet β cells, and the loss of mass gradually exacerbates with the prolongation of T2D and becomes the main factor involved in insufficient secretion of insulin.

Apoptosis due to inflammation, ERS, and oxidative stress has long been thought to be the main cause of β cell dysfunction. However, in recent years, dedifferentiation, senescence and other forms of cell death, such as autophagy, pyroptosis and ferroptosis, have been found to be involved in the loss of β cells.^113–117^ The Aldehyde dehydrogenase 1 isoform A3 (ALDH1A3), normally not expressed in mature islets, is activated in diabetic patients, which damages characterization of β cells, leads to dedifferentiation and impairs secretory function.^118^

#### Insulin synthesis and secretion pathways

The insulin gene is transcribed to mRNA with the aid of various transcription factors such as MAF bZIP transcription factor A (MAFA),^119^ and pancreatic duodenal homeobox 1 (PDX1).^120^ Subsequently, the preproinsulin is translated at the cytosolic surface of the ER and then translocated into it. Within the ER, the signal peptide is cleaved, disulfide bonds form, and the peptide is folded. Proinsulin moves into the Golgi body where it is sorted and packed into secretory vesicles. Within these vesicles, the C-peptide is cleaved off, forming mature insulin.^121^ In general, most nutrients that stimulate insulin secretion also increase proinsulin biosynthesis, with glucose the most physiologically relevant one.^122^

In the classical theory of GSIS, the glucose is transported into islet β cell through GLUT2, and generates pyruvate, entering tricarboxylic acid cycle (TCA) to produce ATP. Increased ATP/ADP ratio induces the closure of ATP-sensitive potassium (K_ATP_) channels, leading to membrane depolarization followed by activation of voltage-gated Ca^2+^ channels (VGCC) and Ca^2+^ influx. The increase in Ca^2+^ ultimately activates insulin granular docking and releasing from the “ready releasable” pool^123^ (Fig. 2b).

#### Hyperglucotoxicity and lipotoxicity

Persistent hyperglycemia and excess fatty acids in the circulation due to overnutrition and IR have widespread and significant effects on β cell function and mass. In addition to involving several shared pathways (e.g., inflammation, ERS, and oxidative stress), they have several individual effects.

High glucose stimulates the continuous secretion of insulin. This process does not involve changes in gene expression at first, but results in secretion failure due to physical consumption. As exposure progresses, the expression of the insulin synthesis transcription factors such as PDX1 and MAFA decreases, disrupting the insulin synthesis. What’s worse, patients with T2D were observed a decreased expression of GLUT1/2, which reduce intracellular transportation of glucose, thus producing less ATP and impaired insulin releasing. In addition, MYC, a transcription factor that improves sensitivity to apoptosis stimulation, is upregulated, along with the activation of other apoptotic pathways,^26,124,125^ causing irreversible damage or even death of β cells. It is called glucotoxicity previously,^126^ however, we may call this phenomenon hyperglucotoxicity, as a normal glucose level is not toxic to islet β cells.

Lipotoxicity is the effect of abnormal lipid metabolism on islet β cells. Excess NEFAs, which stimulate the secretion of insulin, disrupt the function of β cells by increasing basal insulin levels but inhibit GSIS.^127^ SFAs, such as palmitic acid, can increase the production of ceramide and NO, leading to impaired insulin synthesis, and the apoptosis of β cells through inflammation, ERS, and oxidative stress, which can be blocked by unsaturated fatty acids.^127,128^

Lipoproteins and cholesterol have also been found to impact β cell function and mass. Low-density lipoprotein (LDL) can promote apoptosis, while high-density lipoprotein (HDL) can block it.^129^ In addition, the cholesterol homeostasis of β cells is essential for the insulin secretion, as accumulated cholesterol in the plasma membrane resulting in impaired secretion via exocytosis, and reduced cholesterol in insulin granules making them enlarged, also resulting in secretory dysfunction.^130,131^ Many polymorphisms and mutations in the genes involved in cholesterol homeostasis of β cells are associated with T2D in multiple ethnic populations.^129^

#### Inflammation

The inflammatory response in islets can be secondary to a variety of factors, such as metabolites, ROS,^132,133^ and proinflammatory factors released by adipose tissue with low-grade chronic inflammation.^30,31^ These compounds induce an increase in cytokines such as IL-1β, activating NF-κB signaling,^134^ leading to IL-1β-mediated β cell dysfunction and apoptosis.^134^ The NLRP3 is also upregulated resulting from activated NF-κB, and activates caspase 1 which induces β cell pyroptosis, a type of programmed cell death characterized by the formation of pores in the plasma membrane, leading to the release of proinflammatory cytokines and subsequent cell lysis^38,135^ (Fig. 2c).

In addition, increasing STAT3 from activated JAK/STAT signaling pathway might be involved in the apoptosis of β cell through PTEN, which inhibits Akt cascade, resulting in exacerbated hyperglycemia^136^ (Fig. 2c).

Immune cells in islets also contribute to the loss of β cells. Macrophages induce the expression of the neutrophil chemokine (C-X-C motif) ligand 8a (Cxcl8a) in β cells under ERS, recruit neutrophils into islets thus promoting the loss of β cells.^137,138^

#### ERS

Continuous insulin production and secretion in β cells under chronic hyperglycemia and IR result in the overload of protein folding machinery and the accumulation of excessive misfolded or unfolded proteins in the ER, therefore triggering UPR and the ERS. In addition, many metabolites and also ROS are capable of activating ERS networks directly.^139,140^

The UPR relieves cellular load by reshaping the capacity for ER synthesis. However, sustained activation of the UPR can trigger the apoptotic pathway, leading to apoptosis of β cells.^141,142^ ERS-induced apoptosis in β cells is largely dependent on increasing CHOP and decreasing BCL-2, while activation of IKKβ and JNK1 by IRE1α binding with TRAF2 also promotes mitochondria-mediated apoptosis in β cells^37,58,59^ (Fig. 2d).

Hyperglucotoxicity and lipotoxicity increase the expression of islet THADA. Firstly, it reduces ER calcium storage by reducing calcium reuptake and increasing calcium leakage. Secondly, the decreasing insulin 1/2 (INS1/2) transcription and the smaller insulin-secreting vesicles are observed after the upregulation of THADA, together leading to the impaired insulin secretion. In addition, THADA can cause apoptosis in β cells by activating the death receptor 5 (DR5)/Fas-associated death domain (FADD)/caspase 8 pathway under ERS.^143^

#### Oxidative stress

ROS are byproducts of mitochondrial oxidative phosphorylation and are scavenged by antioxidant systems.^125^ The expression of the antioxidant enzymes in islet β cells is low, leading to a greater tendency for them to be affected by oxidative stress.^125,139^

Under chronic hyperglycemia, glycolysis becomes saturated, and excessive ROS are produced by alternative metabolic pathways to induce oxidative stress, causing mitochondrial and DNA damage. The impaired mitochondrial fails to produced sufficient ATP in a short time to activate VGCC, leading to impaired release of insulin granular,^123^ while the downregulation of PDX1 and MAFA results in reduced transcription of insulin.

In addition, excessive NEFAs lead to increasing lipid esterification and ceramide production, causing oxidative stress, and promoting the production of NO. It also downregulates PDX1 and MAFA,^26^ thus resulting in impaired insulin synthesis^63,139^ (Fig. 2c).

Furthermore, JNK activated by ROS under chronic hyperglycemia also leads to mitochondrial-induced apoptosis via multiple genes regulating cell proliferation and survival such as BCL-XL and BCL-2^80^ (Fig. 2d). MST1 in the hippo pathways is also strongly activated in T2D, inducing β cell apoptosis through activation of JNK and impairing β cell function through destabilizing PDX1^144^ (Fig. 2i). Mature islet β cells lack the expression of YAP, while overexpression of YAP in islets is associated with proliferative and antiapoptotic effects.^145^

#### Ectopic lipid deposition in islet

Nonalcoholic fatty pancreas disease (NAFPD) is defined as fat accumulation in the pancreas associated with obesity and the MDS.^146^ A 10-year prospective cohort study showed an independent association between fatty pancreas and subsequent T2D development.^147^

Obesity and IR lead to an increased delivery of NEFAs from adipose tissue to the liver and pancreas. However, unlike in hepatic tissue, fat deposition in pancreatic tissue occurs not intracellularly but rather intercellularly via adipocyte infiltration of the intralobular region, in both acinar and islet cells.^148^ The increased LDL, lipocalin-2, and hepatokine fetuin, as well as reduced adiponectin, may promote fat accumulation in the pancreas, which might be enhanced by inflammation and oxidative stress through the serine/threonine protein kinase 25 pathway.^146^

Although lack of exhaustive research on mechanism, fat accumulation in the pancreas is associated with β cell lipotoxicity, which may be enhanced from local paracrine pathways.^149^ It results in β cell dysfunction,^150^ leading to the initiation of a vicious cycle that further aggravates metabolic diseases and NAFPD.

#### Islet amyloid polypeptide (IAPP)

IAPP, a protein that is coexpressed and cosecreted with insulin by β cells, forms membrane-permeable toxic oligomers and accumulates in the ER under continuous demand for insulin.^151,152^ The mechanism of IAPP-induced cell death may also include inflammation and oxidative stress.^153^

As a compensatory mechanism, IAPP may activate HIF-1α/6-phosphofructo-2-kinase/fructose-2,6-biphosphatase 3 (PFKFB3) to protect against oligomer toxicity, thus delaying the loss of β cells but at the cost of impairing β cell function.^154^ However, HIF-1α in islets is decreased in T2D, by hyperglycemia through inhibiting the HIF-1α-HIF-1β dimer formation, and by NEFAs through increasing the proteolysis, leading to inability to initiate hypoxic adaptive responses.^155^ Depletion of HIF-1α shows impaired GSIS, due to the decreased basal and glucose-stimulated ATP concentration^155,156^ (Fig. 2h).

#### Other signaling pathways involved in β cell dysfunction

mTOR can phosphorylate AGC family of protein kinases, regulating cell survival and proliferation.^79^ Chronic hyperglycemia activates mTORC1 and reduces mTORC2 activity^157^ in islet β cells, which leads to the translocation of FOXO1 to nucleus and less nuclear PDX1, resulting in reduced insulin synthesis and secretion and impaired β cell mass^80,158^ (Fig. 2e).

Generally, canonical Wnt cascades such as Wnt3a can stimulate insulin secretion and enhance the proliferation of β cell by activating β-catenin/TCF/LEF-mediated Cyclin D1 transcription.^50^ However, in T2D, most non-classical Wnt ligands such as Wnt4 and Wnt2a are highly upregulated,^159^ inhibiting Wnt3a and promoting β cell dysfunction.^160^ In addition, Wnt2b and TCF7L2, encoding an important transcription factor TCF4, are associated with the susceptibility of T2D among individuals with impaired glucose tolerance^161^ (Fig. 2g)

Notch signaling is also critical for pancreas development and endocrine specification. Hyperglycemia increases notch activity in vitro and vivo, and consistent activation of notch results in impaired GSIS and blocks maturation of β cells, which may be a result of degradation of MAFA due to the blockage of its binding to kat2b.^162^ In addition, activated notch may affect islet architecture through Ephrin A5 (EFNA5) which is increased in T2D^163^ (Fig. 2e)

**Fig. 3:**
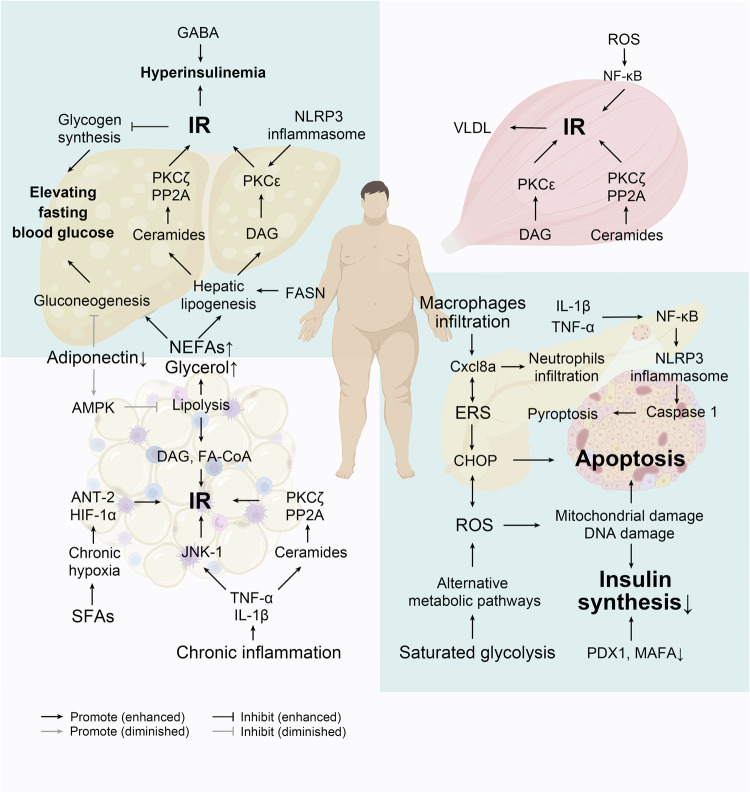
A sketch of pathogenesis of T2D. Briefly, Under the stimulation of SFAs, hypoxia in adipocytes due to enhanced SFAs metabolism, and the release of inflammatory factors from macrophage infiltration cause chronic inflammation in adipose tissues, leading to adipocyte IR and increased lipolysis. NEFAs and glycerol from lipolysis causes increased lipid synthesis, gluconeogenesis, and ectopic fat deposition in the liver, which affects IRS-1/PI3K/Akt2 phosphorylation on the one hand, and increases hepatic release of glucose on the other. At the same time, ectopic fat deposition also occurs in the muscle and leads to elevated circulating VLDL, further exacerbating lipid metabolism disorders and IR in the liver. Persistent hyperglycemia and excess fatty acids in the circulation due to overnutrition and IR further damage β-cell function and mass by some shared pathways, involving inflammation, ERS, and oxidative stress. IR insulin resistance, SFAs saturated fatty acid, NEFAs non-esterified fatty acids, TNF-α tumor necrosis factor α, IL-1β interleukin-1β, JNK c-Jun N-terminal kinase, FASN fatty acid synthase, DAG diacylglycerol, FA-CoA Fatty acyl-coenzyme A, ANT-2 adenine nucleotide translocase 2, HIF-1α hypoxia-inducible factor 1 alpha, NLRP3 NOD-like receptor family pyrin domain-containing 3, GABA γ-aminobutyric acid, PP2A protein phosphatase 2A, PKC protein kinase C, AMPK adenosine 5’-monophosphate-activated protein kinase, VLDL very-low-density lipoprotein, ROS reactive oxygen species, NF-κB nuclear factor-kappa B, Cxcl8a neutrophil chemokine (C-X-C motif) ligand 8a, ERS endoplasmic reticulum stress, CHOP C/EBP homologous protein, PDX1 pancreatic and duodenal homeobox factor-1, MAFA MAF bZIP transcription factor A

Since the diagnosis of T2D relies primarily on exclusion, the clinical phenotype varies widely. However, IR and secondary β cell dysfunction leading to increasing blood glucose accounts for the majority of cases. Elevated blood glucose triggers hyperglucotoxicity, leading to aggravation of IR and deterioration of β cell function, creating a vicious cycle that leads to further elevation of blood glucose to diabetic levels and even hyperglycemic crises.^164^ In addition, hyperglycemia and other metabolic disorders such as dyslipidemia, inflammatory factors, excess IAPP and alterations in the gut microbiota, combine to contribute to IR and/or β cell dysfunction through the common pathways of inflammation, ERS and oxidative stress (Fig. 3).

## Diagnosis and screening

The diagnostic criteria for diabetes are internationally consistent. Both the ADA and the World Health Organization (WHO) have adopted fasting blood glucose (FBG), 2-hour postprandial blood glucose (2-h PG), and random blood glucose levels as diagnostic criteria for diabetes mellitus; later, HbA1c was included in the diagnostic criteria for expanded screening. The diagnostic cutoff points are shown in Table 1. And recently, International Diabetes Federation (IDF) state that people with 1-hour postprandial glucose (1-h PG) ≥8.6 mmol/L are considered to be have prediabetes while ≥11.6 mmol/L to have diabetes.^165^ However, for prediabetes, which is defined as impaired fasting glucose (IFG) or impaired glucose tolerance (IGT), the diagnostic scope of the WHO and ADA for FBG and HbA1c are not completely consistent. In addition, the WHO divides individuals with prediabetes into two groups (IFG or IGT) without distinguishing between those with both IFG and IGT (IGF + IGT), while the ADA divides those individuals into three groups (IFG, IGT, IGF + IGT). Our meta-analysis revealed that patients with prediabetes and IGF + IGT have a greater risk of T2D, suggesting that it is more reasonable to classify prediabetes into three subtypes, as ADA does.^166^ T2D has long been found mostly in adults, but it is increasingly common in adolescents and children due to the increase in obesity in these individuals.^167^ It is important to note that we set absolute blood glucose cutoff points, but blood glucose abnormalities and the risk of developing T2D are continuous processes. A meta-analysis of 16 studies showed that for people with HbA1c between 5.5–6%, the 5-year risk of developing diabetes is 9–25%, while for people with HbA1c between 6–6.5%, the 5-year risk increases to 25–50%.^168^ The Whitehall II cohort study showed a linear trend in glycemic trajectories during the natural onset of T2D, with a sharp increase in blood glucose 2 years before T2D.^169^ Baseline characteristics at diagnosis of T2D might be used to predict early glycemic deterioration followed by more aggressive interventions. A study revealed that baseline characteristics such as younger age (<58 years), higher HbA1c, fasting glucose, and TG levels were associated with subsequent glycemic control, and patients with younger age and higher HbA1c at baseline were more likely to fail to maintain HbA1c <7%,^170^ which indicates the crucial in early diagnosis.

**Table 1:** Criteria for the diagnosis of diabetes and prediabetes

	ADA^1^	WHO^353^
Diabetes	FPG ≥7 mmol/L	
	2-h PG during OGTT ≥11.1 mmol/L	
	RPG ≥11.1 mmol/L	
	HbA1c ≥6.5%	
Prediabetes	FPG 5.6–6.9 mmol/L	FPG 6.1–6.9 mmol/L
	2-h PG during OGTT 7.8-11.0 mmol/L	
	HbA1c 5.7–6.4%	HbA1c 6.0–6.4%

*ADA* American Diabetes Association, *WHO* World Health Organization, *FPG* fasting plasma glucose, *2-h PG* 2 h-plasma glucose, *OGTT* oral glucose tolerance test, *RPG* random plasma glucose, *HbA1c* glycated hemoglobin

In 2021, it is estimated that almost one in two adults with diabetes were unaware of their diabetes status, with nearly one-quarter of those with diabetes diagnosed in North America and the Caribbean, 52.8% in the Western Pacific, 51.3% in Southeast Asia and 53.6% in Africa remaining undiagnosed.^1,171^ Because it is asymptomatic, patients usually cannot notice. However, the risk of diabetes still exists, and the duration of glycemic burden is a strong predictor of adverse outcomes.^1^ Therefore, screening for diabetes is necessary and crucial, especially for patients with high-risk factors, and early diagnosis and treatment can effectively improve patient prognosis. Age, preobese/obese status and prediabetes are the major risk factors for diabetes. The risk of diabetes increases significantly with age, and it is recommended by the ADA that all people ≥35 years old be screened for diabetes since the positive detection rate of diabetes is greatly increased.^1^ Preobesity/obesity is defined by body mass index (BMI), which is considered preobesity if the BMI is ≥25 kg/m^2^ (≥23 kg/m^2^ in Asia) or obese if the BMI is ≥30 kg/m^2^ (≥27.5 kg/m^2^ in Asia). Additionally, preobesity/obesity can cause hypertension, IR and lipid disorders, which all contribute to T2D and are used for T2D screening.^172^ However, BMI cannot be used as a proxy for the amount of body fat since people with the same BMI may have different fat contents and distributions, which represent different risks of diabetes.^173^ Several indicators have been proposed to redefine obesity rather than BMI. The waist-to-height ratio (WHtR), defined by waist circumference (cm)/height (cm), has been proposed as a surrogate indicator of BMI. A meta-analysis consisting of 72 prospective studies showed that central adiposity, defined as the accumulation of excess fat in the abdominal area, was positively associated with all-cause mortality, and the WHtR had the highest hazard ratio (1.24), suggesting that central adiposity measurements, such as the waist-to-hip ratio, the WHtR, and waist circumference, can be used as supplements for determining BMI.^174^ According to the guidelines of the National Institute for Health and Care Excellence (NICE), adults with a BMI <35 kg/m^2^ are also recommended for whom the WHtR is measured to assess central adiposity and central adiposity and to use these data to help assess and predict health risks.^175^ The International Society of Hypertension (ISH) also recommends that body weight be managed by maintaining the WHtR below 0.5, which is suggested to be the cutoff point by the NICE.^176^ In addition to risk factors, the practical way to screen for diabetes is to perform fasting blood glucose and HbA1c tests at the same time as annual physical examination, which can detect the vast majority of diabetic patients in time. Our study revealed that a point-of-care test (POCT) of FBG ≥5.3 mmol/L is a more cost-effective method for screening those with undiagnosed diabetes, suggesting that patients with a POCT of FBG ≥5.3 mmol/L should perform confirm test of diabetes. We believe that this method is feasible and inexpensive for detecting diabetes and is worth recommending because POCT is convenient and readily available.^177^

For many years, venous blood glucose and laboratory-based HbA1c have been used as standard biomarkers and technologies for diagnosing and screening of diabetes. Meanwhile, novel potential biomarkers are still being sought.

Some of the clinically validated biomarkers such as glycated albumin (GA), fructosamine (FA), 1,5-anhydroxybergamottinol (1,5-AHG) showed high specificity and sensitivity in diagnosis and can be used for alternative markers when HbA1c cannot be measured in specific situations.^178^ In addition, there are a number of novel markers such as fetuin-A, branched-chain amino acids (BCAAs), adipokines, linoleoylglyglycerophosphocholine (L-GPC), lysophosphatidylcholine (LysoPC) which are discovered by metabolomics showed strong relationship with HbA1c or blood glucose, indicating their future potential in diagnosis and screening.^178,179^ More than 30 genetic factors are associated with increased risk for T2D. However, the risk alleles in these loci all have relatively small effects and do not significantly enhance our ability to predict the risk of T2D.^180^

Unconventional body fluid glucose testing will be a new direction for future development, but further studies are still needed to support its clinical application. There have been reports that glucose in sweat, tears, and saliva for diagnosis and screening of diabetes.^181–183^ Study showed that glucose levels in saliva are highly significantly correlated with serum glucose levels, but there is a lack of validated methods to detect salivary glucose nor have critical reference values been established, and the relationship between glucose in tears and sweat and serum glucose is inconclusive.^178,180^ In addition, urine glucose is advocated by the IDF as a screening test for undiagnosed diabetes in a low-resource setting without other procedures.^178,180^

Portable blood glucose testing and continuous glucose monitoring (CGM) are also commonly used as blood glucose testing devices. Current evidence is insufficient to support their use for diagnostic purposes, however, there have been results showed its potential. 40% and 3% patients who would have been considered to have normal FBG based on the first FBG measurement could been reclassified as having glucose in the prediabetes and diabetes ranges, respectively, based on sequential measurements throughout the study.^184^ CGM detect higher blood glucose levels in pregnant women with gestational diabetes mellitus (GDM) prior to the OGTT, suggesting a potential future role in the early diagnosis of GDM.^185^

POCT devices are convenient and fast, and POCT HbA1c have been allowed for diabetes screening and diagnosis but should be restricted to U.S. Food and Drug Administration (FDA)-approved devices at CLIA-certified laboratories that perform testing of moderate complexity or higher. Noninvasive glucose sensing is also a promising technique but FDA hasn’t approved any noninvasive device for clinical measurement due to the lack of the ability to produce stable calibration functions required for practical clinical operation.^180^

## Prevention

The Whitehall II study showed that people with prediabetes are already at a steep end of the glycemia trajectory,^169^ which means that blood glucose is likely to increase at an accelerated rate and progress to T2D. In addition, people with prediabetes already exhibit a loss of islet β cells and impairment of target tissues. Approximately 30–50% of islet β cell function is lost in individuals with prediabetes^,^^186^ and approximately 10% of people with prediabetes develop DR or polyneuropathy.^187,188^ Therefore, aggressive actions are necessary and important for preventing T2D in people at high risk (such as those with an HbA1c >6%).

### Lifestyle intervention

Several major trials have shown some effective interventions to prevent the occurrence of T2D and even reduce the risk of TOD. Intensive lifestyle interventions, including physical activities and individualized medical nutrition treatment (MNT), have been proven to be highly effective at preventing diabetes. Exercise, diet or a combination of both can reduce T2D risk by 30–60% according to large-scale RCT trials (the IDPP, DPP, FDPS, and Da Qing trials), and the variation in the reduction rate may be due to differences in the population in those trials, while people with a greater BMI and older age had a greater reduction in T2D risk.^189–192^ Weight loss is an important factor in intensive lifestyle intervention since both the DPP and FDPS trials set a goal for weight loss achieved by physical activities and a calorie-reduced diet. As shown in the DPP, for every kilogram of weight loss, the risk of T2D was reduced by 16%.^193^ There is also evidence that physical activity and nutrition have preventive effects and metabolic improvement independent of body weight. In the Da Qing trial, analysis of the relatively lean subgroup (BMI <25 kg/m^2^) showed that even with weight gain, lifestyle intervention still significantly reduced the risk of T2D.^189^ In the long-term follow-up, both physical activity and nutritional treatment showed sustained benefits for T2D prevention, which still significantly reduced the risk of progression to T2D by 45% and 39%, respectively, after 23 and 30 years of follow-up in the Da Qing trial; by 43% after a 7-year follow-up in the FDPS; and by 27% after a 15-year follow-up.^194–197^ Da Qing trials also showed significant cardiovascular benefits, which included a reduced incidence of cardiovascular events and cardiovascular and all-cause mortality; however, follow-up with DPP did not reveal improvements in microvascular or cardiovascular mortality.^194,195,197,198^

### Medications

There are no medications have been proven for a specific indication of T2D prevention by FDA; however, several hypoglycemic agents metformin, α-glucosidase inhibitors (AGIs), thiazolidinediones (TZDs), sodium-glucose cotransporter inhibitors (SGLT-is, classified into two categories: SGLT-2i and SGLT-1 and SGLT-2 dual inhibitors, SGLT-1/2i), and glucagon-like peptide-1 (GLP-1) (we refer to this name, because its active ingredient is GLP-1, which includes human GLP-1 analogues, animal-origin GLP-1 or derivatives of exantine-4, while GLP-1 receptor agonists, which are now usually called, is the action target instead of active ingredients, and does not conform to the traditional classification by chemical name.) have been shown to prevent T2D in studies. Metformin was the first hypoglycemic agent studied for its ability to reduce T2D risk and it reduced the T2D risk by 31% and 26.5% in the DPP and IDPP trials, respectively. Acarbose reduced the risk of T2D by 25% in the STOP-NIDDM trial.^199^ Neither metformin nor acarbose has any cardiovascular benefits. TZDs reduced the risk of T2D by 60% and 72% in the DREAM and ACT NOW trials, respectively.^200,201^ The IRIS trial randomized nondiabetic patients with a history of recent ischemic stroke or transient ischemic attack and IR to pioglitazone or placebo; the results showed that pioglitazone reduced the risk of T2D by 52% and reduced the risk of any stroke or ischemic infarction by 24%.^202,203^ Those trials suggest that TZDs are comparable or even superior to lifestyle interventions. Pioglitazone not only is effective at preventing diabetes but is also the first hypoglycemic agent shown to have an anti-atherosclerotic effect on cardiovascular disease (secondary prevention) in nondiabetic patients. Liraglutide 3 mg per day significantly reduces the risk of T2D by 80% in individuals with prediabetes and preobesity or obesity.^204^ Another two GLP-1 agents, semaglutide and tirzepatide, significantly convert abnormal glycemia in individuals with prediabetes with preobesity or obesity to normoglycemia.^205,206^ Furthermore, 2.4 mg of semaglutide per week has been shown to reduce the risk of the composite outcome of cardiovascular death, nonfatal myocardial infarction (MI) and stroke (i.e., major adverse cardiovascular events [MACE] by 20%, and the risk of diabetes by 73% in nondiabetic patients with established atherosclerotic cardiovascular disease (ASCVD) and obesity, suggesting cardiovascular benefits (secondary prevention) of semaglutide in nondiabetic people and prevention of T2D.^207^ However, GLP-1 has been demonstrated in only a specific population with preobesity or obesity, and further evaluation of the prevention of T2D in the general population is needed. The SGLT-2i dapagliflozin demonstrated a 33% reduction in the risk of T2D in a pooled analysis of the DAPA-CKD and DAPA-HF trials.^208^ In the DAPA-MI trial, which enrolled nondiabetic patients with acute MI, dapagliflozin also significantly reduced the risk of diabetes.^209^

Metformin is well studied and has the longest history of safety for the prevention of diabetes and is recommended by ADA for patients at high risk of T2D; however, in terms of the chances of reducing the risk of diabetes, it is not as effective as liraglutide, semaglutide or TZDs, although there are no head-to-head studies. In addition, there is no evidence that metformin reduces the risk of ASCVD as pioglitazone or semaglutide did. Therefore, we do not consider metformin to be a reasonable choice for the prevention of T2D in terms of its cardiovascular effect and preventative effect diabetes. For patients without severe osteoporosis and heart failure (HF), particularly for ASCVD patients with IR, pioglitazone is a reasonable choice for the prevention of T2D. For preobese or obese people, especially those with ASCVD, semaglutide and liraglutide are reasonable choices. Dapagliflozin may be a reasonable choice for patients with HF and/or CKD.

## Treatment

Patients with T2D often exhibit a variety of comorbidities, such as dyslipidemia, preobesity/obesity, CKD and cardiovascular disease (CVD), and suffer from TOD caused by MDS. Therefore, T2D treatment usually includes holistic management of MDS, such as MASLD, hyperglycemia, dyslipidemia, hypertension, and preobesity/obesity, which are all risk factors for CVD and CKD by lifestyle intervention and necessary medication to reduce TOD, improve cardiovascular and renal outcomes, span life expectancy and improve quality of life. Notably, due to the diabetes stigma in media and society, for example, attributing diabetes simply to a personal unhealthy diet and ignoring genetic, regional, economic, and social factors, diabetic patients do not get support they deserve and may suffer from psychologically and socially negative impacts. Therefore, mounting voices have called for society to reduce diabetes stigma and patients to strengthen awareness of diabetes to avoid self-stigma.^210^

Since the development of hypoglycemic agents, there have been various drugs that act via different mechanisms, ranging from the older agents metformin, insulin secretagogues, AGIs, TZDs, and insulin to new ones, such as dipeptidyl peptidase-4 inhibitors (DPP-4i) and GLP-1; SGLT-is, which includes SGLT-2i, such as dapagliflozin and empagliflozin; and SGLT-1/2i, such as sotagliflozin and canagliflozin. How to choose the appropriate hypoglycemic regimen for patients has become an increasingly prominent need. It is reasonable to stratify treatment regimens according to glycemic targets and current blood glucose levels and based on this, personalized treatment considering target organ protection, comorbidities, treatment goals, costs, access to medication and patient preferences can be administered.^211^

### Stratified glycemic targets

Substantial studies have shown that good glycemic control can reduce TOD and cardiovascular risk. The glycemic targets should be specialized according to individual characteristics (duration of diabetes, risk of hypoglycemia, advanced age, microvascular disease and comorbidities such as CVD, life expectancy, and social factors). For people with diabetes, in general, it is appropriate for most nonpregnant adults to have their glycemic target controlled at less than 7%. For young or middle-aged patients without organ lesions, the HbA1c level should be reduced to normal or <5.7% to minimize vascular lesions due to a long life expectancy.^1^ Patients with diabetes who have a relatively long enough life expectancy to achieve macro- or microvascular benefits of tighter glycemic control and few microvascular diseases and comorbidities can pursue more stringent glycemic control (<6–6.5%^1^) without increasing hypoglycemic risk. For those with advanced age, short life expectancy, frequent hypoglycemia and severe comorbidities, more relaxed targets should be taken (<8% recommended by the ADA^1^ and 8.5% recommended by the European Society of Cardiology (ESC)^212^) because the risks associated with intensive glycemic control may outweigh the benefits. Several key trials suggest that although intensive glycemic control may reduce the incidence of macrovascular and microvascular complications,^213,214^ it is not significant for patients with longer durations of diabetes, advanced age and established or at high risk of CVD,^215^ and even showed increased mortalities as ACCORD trial was halted early because of increased mortality rate.^216^

### Baseline HbA1c stratification

Treatment regimens need to be stratified based on current HbA1c and target HbA1c, as summarized in Table 2. For those T2D patients with a present HbA1c level ≤ the target of HbA1c + 0.5%, lifestyle interventions alone can be reasonable,^211^ and due to the closer proximity to the glycemic target, lifestyle interventions are sufficient to maintain the target without additional pharmacotherapy. For those with a present HbA1c level > target HbA1c + 0.5% but ≤target HbA1c + 1.5%, lifestyle intervention plus treatment with one type of antidiabetic medication should be considered. For those with a present HbA1c > target HbA1c + 1.5% but ≤10%, lifestyle intervention plus treatment with two types of antidiabetic medications can be considered to achieve glycemic targets more rapidly. When the HbA1c level is >10% or there are symptoms of hyperglycemia or ketonemia, intensive therapy with insulin should be considered to minimize the risk of hyperglycemic crisis.^1,211^

**Table 2:** Stratification of baseline HbA1c and treatment regimens

Baseline HbA1c stratification	Treatment regimens
Present HbA1c ≤ target HbA1c + 0.5%	Lifestyle intervention
Target HbA1c + 0.5% < present HbA1c ≤ target HbA1c + 1.5%	Lifestyle intervention + 1 hypoglycemic agent
Target HbA1c + 1.5% < present HbA1c ≤ 10%	Lifestyle intervention + 2 hypoglycemic agents with different mechanisms
Present HbA1c > 10% with symptoms of hyperglycemia or ketonemia	Lifestyle intervention + insulin
HbA1c, glycated hemoglobin.	

### Lifestyle intervention

Lifestyle interventions, including physical activity, MNT, and weight management, are the foundation of T2D management. Many studies have shown that lifestyle interventions, such as physical activities and MNT, can reduce HbA1c levels by 0.3–2% and even achieve remission of diabetes, defined as an HbA1c < 6.5%, in the absence of pharmacological intervention.^211,217,218^ Physical activities have been shown to effectively lower blood glucose levels, improve insulin sensitivity and cardiopulmonary function and reduce the risk of cardiovascular events.^219–221^ And there have been results demonstrated that physical activity significantly improve time in range (TIR) and reduce glycemic excursion with no increased risk of hypoglycemia^222^ and reduced the risk of progress to CKD in T2D with preobesity/obesity.^223^ Various types of physical activities, including aerobic exercise, resistance exercise training and even brief standing, walking, or other light physical activities during leisure time, help with glycemia management. At least 150 min of moderate-intensity exercise per week is recommended for adults with T2D, and this exercise should be applied for at least 3 days per week, with no more than 2 consecutive days without activity.^1^ In addition, Moderate to vigorous physical activity in the evening showed the lowest risk of mortality, CVD, and microvascular disease in compared to the morning and afternoon in preobese or obese patients, especially those with T2D.^224,225^ Currently, most guidelines recommend time-based physical activities. A study showed step- and time-based physical activity, are comparable in the association with all-cause mortality and CVD, suggesting a future consideration for step-based physical activity for personalized choice.^226^

By improving dietary structure and energy intake, MNT can improve blood glucose and metabolic parameters and delay or prevent MDS-related TOD. There is no one-size-fits-all dietary pattern for patients with T2D, and it should be individualized based on their metabolic goals and dietary preferences (traditional, religious, cultural, etc.). Several dietary patterns, such as a continuous energy-restricted diet, a Mediterranean diet, a low-carbohydrate or very low-carbohydrate diet, intermittent fasting and time-restricted eating, have been shown to lead to glycemic reduction and metabolic benefits in patients with T2D.^217,227–232^ Recently, a 5:2 intermittent fasting diet showed even better glycemic control in newly diagnosed diabetic patients compared with metformin or empagliflozin.^233^ And high-fiber diet enriches the proportion of beneficial gut microbiota and improved glucose hemostasis, inflammation and emotional mood.^234^

Although there is no single best diet pattern, the key nutritional principles among the variable patterns should be emphasized, including nonstarchy vegetables, whole fruits, legumes, whole grains, nuts/seeds, and low-fat dairy products, as well as minimizing the consumption of meat, sweets, refined grains, and ultra-processed foods in people with prediabetes and diabetes.^1^

Lifestyle interventions are long-term behaviors, and digital apps available on prescription (DiGA) and telemedicine may help with long-term monitoring and improve adherence, and reduce HbA1c by 0.3-0.5% according to a systematic review.^235^

### Choice of pharmacologic therapy

The selection of hypoglycemic agents should consider the three goals of hypoglycemic efficacy, weight management and cardiorenal risk reduction. For patients without established CVD, CKD or multiple high-risk factors, priority can be given to metformin or other hypoglycemic agents that have sufficient glucose-lowering effects to achieve and maintain treatment goals. GLP-1 (4.5 mg dulaglutide, 2.0 mg semaglutide), GLP-1/glucose-dependent insulinotropic polypeptide (GIP) dual receptor agonist (GLP-1/GIP RA) tirzepatide, insulin and combination therapy have been proven to have very high efficacy, while other GLP-1s, metformin, SGLT-is and TZDs have high efficacy, and DPP-4is have intermediate efficacy. For patients without established CVD, CKD or multiple high-risk factors but with weight loss demand, the selection of hypoglycemic agents should consider both glucose-lowering efficacy and weight loss efficacy. Semaglutide and tirzepatide have been proven to be highly effective at decreasing weight. For those with CVD or CKD or with high cardiorenal risk, hypoglycemic agents with additional cardiorenal benefits are recommended in addition to comprehensive cardiovascular risk management. For patients with established ASCVD or at high risk of ASCVD, GLP-1 and SGLT-is agents with proven ASCVD benefits are recommended as the first choice, and TZD is considered a complementary agent when glycemia is still above the target HbA1c after the combination of GLP-1 and SGLT-is. For patients with HF, SGLT-is agents with proven HF benefits are recommended. For patients with CKD, SGLT-is therapy with proven renal benefit is recommended, and GLP-1 could be the choice if SGLT-is is unavailable. A combination of these two agents with proven greater glucose-lowering efficacy is considered to achieve the target glycemic targets.^1^

#### Classification according to the characteristics of hypoglycemic agents

At present, the most commonly used classification for hypoglycemic drugs is based on the drug structure, but this classification lacks clinical practicality. Our team classified hypoglycemic agents in detail based on their hypoglycemic mechanisms^236^(Table 3) and organ protective effects, which is helpful for drug selection and personalized treatment in clinical practice, as Table 4 shows.

**Table 3:** Targets and mechanisms of hypoglycemic agents

Medicines	Target organs	Action target	Mechanisms of hypoglycemia
Metformin	Liver, muscle adipose	Modulate mitochondrial enzymes and hepatic redox state, and increases cellular AMP kinase	↓hepatic gluconeogenesis; ↑glucose uptake
SUs	Pancreatic islet β cell	Bind to SUR-1 subunit of the K-ATP channels, leading to channel closure and membrane depolarization	↑insulin secretion
Meglitinides or glinides	Pancreatic islet β cell	Bind to SUR-1 subunit of the K-ATP channels, leading to channel closure and membrane depolarization	↑insulin secretion
AGI	Intestine	Inhibit α-glucosidase in the small intestinal brush border	↓absorption of complex polysaccharide
TZD	Muscle, adipose	Activate PPAR-γ to increase adiponectin and GLUT-4 expression while inhibiting TNF-α effect in adipocytes	↑fatty acid uptake and storage; ↓fat accumulation in the liver, muscle, and pancreas; ↑glucose uptake
DPP-4i	Pancreatic islet α/β cell	Block degradation of incretin hormones (GLP-1 and GIP) by the enzyme DPP-4 and increase incretin hormones levels	↑insulin secretion; ↓glucagon secretion
GLP-1	Pancreatic islet α/β cell	Resistant DPP-4 to extend the half-life of GLP-1 simulate GLP-1 receptor along with “supra-physiologic” GLP-1 levels	↑insulin secretion; ↓glucagon secretion
SGLT-is	Kidney, intestine	Block SGLT-1 in intestine or/and SGLT-2 in kidney receptors and lower the renal threshold for glycosuria	↓intestinal or/and renal glucose reabsorption

*SUs* Sulfonylureas, *SUR-1* sulfonylurea receptor 1, *K-ATP* ATP sensitive potassium, *AGI* α-glucosidase inhibitors, *TZD* thiazolidinediones, *PPAR-γ* peroxisome proliferator-activated receptor-γ, *GLUT-4* glucose transporter type 4, *TNF-α* tumor necrosis factor, *DPP-4i* dipeptidyl peptidase-4 inhibitors, *GIP* glucose-dependent insulinotropic polypeptide, *GLP-1* glucagon-like peptide-1, *SGLT-is* sodium-glucose cotransporter inhibitors

**Table 4:** Classification of hypoglycemic agents based on hypoglycemic mechanism and organ protective effects

Treatment needs	Classification by mechanism of hypoglycemia or target organ protection or safety	Medications
HYPOGLYCEMIC EFFECT	Dependent on pancreatic islet β cell function	Secretagogues
	Partly dependent on pancreatic islet β cell function	DPP-4i, GLP-1
	Independent of pancreatic islet β cell function	Metformin, AGI, SGLT-is, TZD, Insulin
CV BENEFITS	Glucose-dependent benefits	Secretagogues, DPP-4i, insulin
	Glucose-independent benefits	Pioglitazone, SGLT-is GLP-1: dulaglutide, semaglutide (SQ), liraglutide
	Possible glucose-independent benefits	Metformin, AGI
RENAL BENEFITS	Glucose-dependent benefits	Secretagogues, DPP-4i, insulin
	Glucose-independent benefits	SGLT-is GLP-1: dulaglutide, semaglutide (SQ), liraglutide
IMPACT ON BODY WEIGHT AND VISCERAL FAT	Weight-loss effect	Metformin, AGI, GLP-1, SGLT-is
	Weight-neutral effect	DPP-4i
	Weight gain but visceral fat loss	TZD
	Weight gain in non-overweight or non-obese patients	Secretagogues
	Weight-gain effect	Insulin
SAFETY BASED ON HYPOGLYCEMIC RISK	No significant hypoglycemic risk	Metformin, AGI, TZD, SGLT-is
	Low hypoglycemic risk	DPP-4i, GLP-1
	Moderate hypoglycemic risk	Secretagogues, basal insulin
	High hypoglycemic risk	Insulin (except for basal insulin)
SAFETY BASED ON IMPACT ON LIVER FUNCTION	Safe in mild to moderate liver function impairment	All hypoglycemic medications
	Safe in severe liver function impairment	Linagliptin, lixisenatide, dulaglutide, insulin

This table is modified according to Zhang et al.^211^

*DPP-4i* dipeptidyl peptidase-4 inhibitors, *GLP-1* glucagon-like peptide-1, *AGI* α-glucosidase inhibitors, *SGLT-is* sodium-glucose cotransporter inhibitors, *TZD* thiazolidinediones, *CV* cardiovascular, *SQ* subcutaneous

#### Initial selection of hypoglycemic drugs for newly diagnosed patients with different characteristics

##### Hypoglycemic agents for T2D patients with preobesity/obesity

Weight loss is an important component of glycemic management and can significantly reduce blood glucose levels and the risk of TOD, and a 5–10% weight loss in T2D patients may decrease the HbA1c by 0.6-1.0% and improve metabolic status and cardiovascular risk factors.^237,238^ Currently, there are voices suggesting that weight loss be a primary target treatment rather than just a strategy for lowering HbA1c since weight loss has cardiovascular benefits independent of blood glucose.^239,240^ The effect of hypoglycemic agents on body weight should be considered, and it is reasonable to use drugs with weight-loss effect in T2D patients with preobesity/obesity as an adjunct to lifestyle interventions. The newer drugs GLP-1/GIP RA and GLP-1 have shown substantial and clinical reductions in body weight in key trials. In the STEP-2 and SCALE trials, semaglutide 2.4 mg and liraglutide 3.0 mg reduced body weight by 9.6% and 6%, respectively, in preobese and obese T2D patients.^241,242^ Besides, in STEP-HFpEF DM trial, compared with placebo, T2D patients with obesity-related HF received semaglutide 2.4 mg showed a 6.4% reduced mean weight and 7.3 points increase of Kansas City Cardiomyopathy Questionnaire clinical summary score (KCCQ-CSS) which mean improved HF-related symptoms, physical function, quality of life, and social function.^243^ In the SURMOUNT-2 trial, GIP/GLP-1 RA tirzepatide demonstrated a greater weight loss of 14.7%; however, semaglutide and tirzepatide were both approximately 5% less effective in preobesity and obese people with T2D than in those without diabetes. This might suggest that weight loss is more difficult in people with diabetes.^205,206,244^ Substantial evidence that GLP-1 weight loss is due in large part to reduced food intake.^245^ Mountains of evidence suggests that multiple sites in the brain are more likely to dominate the effects of GLP-1 in inhibiting intake and reducing body weight, such as increasing preingestive satiation via dorsomedial GLP-1 receptors (GLP-1R) in hypothalamus-NPY/AgRP in arcuate neural circuit and reduce food intake by GLP-1R-mediated suppression of AMPK in hindbrain neurons.^246^ In addition, GLP-1 delays gastric emptying and reduces food intake via vagal GLP-1R.^247^

Recently there has been inconsistency regarding the management of GLP-1 in the perioperative period, with evidence suggesting that GLP-1 use increases gastric residue rates and increases the risk of incomplete procedures and aspiration,^248,249^ but there is also evidence suggesting that this does not increase the risk of postoperative complications such as postoperative pneumonia.^249,250^ And gastric emptying delay of 36 min due to GLP-1 is of limited magnitude relative to standard periprocedural fasting periods.^251^

##### Hypoglycemic agents for T2D patients with MASLD

Nonalcoholic fatty liver disease (NAFLD) is a histologic spectrum of hepatic disorders ranging from steatosis and steatohepatitis to advanced fibrosis, cirrhosis and even increased risk of hepatocellular carcinoma (HCC) and liver-related mortality. NAFLD has been replaced by the term “metabolic dysfunction associated steatotic liver disease (MASLD)” to enhance awareness, understanding of the disease and drug/biomarker development.^252,253^ MASLD and T2D are generally considered to have strong bidirectional relationships both epidemically and pathologically and share a common mechanism, IR.^254^ A large meta-analysis estimated that approximately 56% of T2D patients in the global population have MASLD.^255^ Two meta-analyses suggested that patients with MASLD have a two-fold increased risk of developing diabetes.^256,257^ A recent cohort study showed that regression of MASLD reduced the risk of diabetes; however, the benefits are evident only in patients with low MASLD fibrosis scores, suggesting early intervention for MASLD.^258^

*TZDs*. TZDs, which improve insulin sensitivity, have been tested for their effect on MASLD. In the first RCT to prove the effect of TZDs on MASLD, 55 prediabetic patients or T2D patients with metabolic dysfunction-associated steatohepatitis (MASH) were randomized to pioglitazone or placebo groups for 6 months. The results showed that pioglitazone significantly improved the histologic features of steatohepatitis compared with placebo, and fibrosis scores significantly improved in the pioglitazone group before and after treatment; however, the change from baseline did not significantly differ from that in the placebo group.^259^ Aithal et al. randomized 74 nondiabetic patients with MASH to pioglitazone or placebo groups for 12 months. The results showed that pioglitazone significantly reduced hepatocyte injury and fibrosis scores compared with those of the placebo.^260^ The FLIRT^261^ trial tested another TZD, rosiglitazone, and 63 patients with MASH were randomized to the rosiglitazone or placebo group for 12 months. The results showed that the mean reduction in steatosis and the proportion of patients reaching a >30% reduction in steatosis in the rosiglitazone group were both significantly greater than those in the placebo group. However, there was no difference in fibrosis score between the groups. A multicenter RCT, the PIVENS^262^ trial, randomized 247 nondiabetic patients treated with MASH into the pioglitazone, vitamin E, and placebo groups for 96 weeks. Compared with placebo, pioglitazone significantly reduced the histologic feature scores, and a greater proportion of subjects in the pioglitazone group achieved resolution of MASH. More patients had improved fibrosis and reduced fibrosis scores, but these changes were not significant. In 2016, an 18-month RCT conducted by Cusi et al. included 101 prediabetic T2D patients with MASH and randomized them to pioglitazone or placebo groups. At the end of the treatment, more patients in the pioglitazone groups achieved the primary outcome and resolution of MASH than did those in the placebo group, and pioglitazone significantly reduced the fibrosis scores.^263^ A similar study conducted by Bril et al. randomized 105 T2D patients with MASH to pioglitazone plus vitamin E, vitamin E, or placebo groups for 18 months. The results showed that more patients in the combination group achieved the primary outcome and resolution of MASH than did those in the placebo group, while there was no significant difference between the vitamin and placebo groups, suggesting the beneficial effect of pioglitazone on MASH. However, no significant changes were observed in fibrosis scores, although there was a trend toward more patients in the combination and vitamin E groups having improved fibrosis stages than in the placebo group.^264^ In summary, the beneficial effects of TZDs on the improvement and even the resolution of steatosis and histologic features of MASH were highly consistent, but the effects on liver fibrosis were inconsistent with several studies suggesting no significant improvement in fibrosis with TZDs, possibly because the intervention time was not long enough to produce significant improvement in fibrosis or because of the differences in baseline fibrosis stage between studies. Two meta-analyses suggested that pioglitazone reverses or delays the progression of fibrosis, including advanced-stage fibrosis, which is an independent predictor of liver-related adverse events and mortality in MASLD/MASH patients.^265–268^ Interestingly, Pepa et al. showed that even a low dose of pioglitazone still significantly improved liver steatosis and inflammation.^269^ The benefits of TZDs in MASLD and fibrosis may be the increased adiponectin levels and the adiponectin-mediated effect on insulin sensitivity and hepatic fatty acid metabolism^270^ and the downregulated fibrogenic factors and related pathways, inflammatory pathways like JAK/STAT and NF-κB signaling pathways.^271–273^

*GLP-1*. LEAN trial randomized 52 patients with MASH to the liraglutide or placebo group. The results showed that more patients treated with liraglutide achieved resolution of MASH than did those treated with placebo, but there was no significant difference in fibrosis improvement.^274^ A phase 2 study testing semaglutide enrolled 320 patients with MASH and fibrosis and randomized them to 0.1, 0.2, or 0.4 mg semaglutide group or the placebo group. The results showed that 0.4 mg semaglutide once daily was associated with a significantly greater incidence of MASH resolution without worsening of fibrosis than placebo. However, the improvement in fibrosis between the groups did not significantly differ.^275^ Another smaller phase 2 study enrolled 71 patients with MASH-related cirrhosis testing 2.4 mg semaglutide once weekly did not reveal significant changes in either the resolution of MASH or improvement in fibrosis despite some improvement of noninvasive markers of disease activity.^276^ Tirzepatide is associated with significantly greater proportion of achieving MASH remission with no worsening of fibrosis compared with placebo^277^ in a phase 2 trial, and another phase 2 trial for dual agonism of glucagon receptor and GLP-1R (GCGR/GLP-1RA) survodutide demonstrated similar improvements in MASH.^278^ The effects of GLP-1 on ameliorating hepatic inflammation, steatosis, and injury are thought to be secondary to weight loss and improved hepatocyte lipid synthesis and oxidation.^245,279^

In summary, based on histopathological results, pioglitazone is the most studied agent; it has shown the strongest beneficial effects of hypoglycemic agents and has shown additional hypoglycemic-independent anti-atherosclerotic effects. Although TZDs can increase body weight, weight gain mostly occurs in subcutaneous fat, while visceral fat, which is closely related to metabolic disorders, does not increase or even decreases.^280–282^ Therefore, pioglitazone has been recommended as the preferred drug.^1,283–285^ Liraglutide, which has shown some pathological evidence of beneficial effects on liver fibrosis and glucose-independent anti-atherosclerotic effects, as well as weight loss, is also one of the preferred drugs for treating T2D with MASLD. Other current hypoglycemic drugs lack evidence of their effect on liver fibrosis and are not considered drugs for T2D patients with MASLD.

##### Hypoglycemic agents for T2D patients with established or high risk of CVD

*ASCVD*. CVD are the leading cause of patients with T2D. T2D tends to coexist with metabolic disorders such as dyslipidemia and hypertension, which are risk factors for ASCVD. In addition, T2D itself is a potent and independent risk factor for CVD.

Specific GLP-1 agents have been shown to improve cardiovascular outcomes, and liraglutide, semaglutide (subcutaneous and oral), dulaglutide, and albiglutide consistently reduce the risk of MACE in people with T2D with established CVD or a high risk of CVD. However, lixisenatide in the ELIXA trial did not significantly alter the incidence of MACE, and exnatide in the EXSCEL trial lower the risk of MACE with no significant difference.^286–292^ Overall, GLP-1 reduced the risk of MACE by 14% according to a meta-analysis comprising these trials.^293^

Some TZDs have shown cardiovascular-protective benefits, as pioglitazone reduced the risk of ASCVD in some trials. The PROactive^294^ trial showed that pioglitazone, compared with a placebo, reduced the risk of composite outcomes (all-cause mortality, nonfatal MI and stroke) by 16% in diabetic patients with established ASCVD. COSTA.IT^295^ trial showed that although pioglitazone had no significant difference in total cardiovascular deaths compared with sulfonylureas in primary ASCVD diabetic patients, it significantly reduced the risk of ischemic cardiovascular events by 33%. The IRIS^203^ trial showed that pioglitazone reduced the incidence of any stroke in nondiabetic patients with IR and stroke, suggesting that pioglitazone has glucose-independent cardiovascular benefits. And the benefits might be partly result from decrease in lectin-like oxidized receptor1 (LOX-1) followed by significant declines in cellular ROS and NF-κB signaling pathways.^296^

Emerging evidence has proven that SGLT-is can reduce the risk of adverse cardiovascular outcomes. The EMPA-REG and CANVAS trials showed that the SGLT-2i empagliflozin and SGLT-1/2i canagliflozin reduced the risk of MACE in T2D patients with or at high risk of CVD by 14%, while the SGLT-2i dapagliflozin did not significantly reduce the risk of MACE, possibly due to the difference in population (approximately 60% with no established ASCVD) between previous trials and strict exclusion criteria.^297–299^ However, ertugliflozin in the VERTIS trial did not show superiority to the placebo in T2D patients with established ASCVD.^300^ SGLT-1/2i, sotagliflozin, reduced the risk of MACE by 16% in the SCORED trial in patients with chronic kidney disease and additional cardiovascular risk factors, and it is the only SGLT-i with evidence of a reduced risk of ASCVD-related macrovascular events of MI and stroke.^301^ In addition, SGLT-2i still showed a remarkable cardiovascular benefit in nondiabetic patients, indicating that SGLT-2i had a blood glucose-independent effect on improving cardiovascular outcomes.

Several GLP-1 and SGLT-is proven cardiovascular benefits are recommended by the ADA to be the first-line hypoglycemic agents for patients with T2D with established ASCVD or at high risk of ASCVD, and combination therapy comprising SGLT-is and GLP-1 may provide additional cardiovascular benefits. Pioglitazone is recommended as a second-line hypoglycemic therapy when HbA1c is still above the target level.^1^ The ESC also suggests that pioglitazone should be considered for reducing ASCVD risk in patients with T2D with ASCVD based on the data and net benefit-risk assessment.^212^

*HF*. Patients with diabetes are twice as likely to develop HF, which leads to increased mortality.^302^ SGLT-is showed a more robust and consistent effect on reducing hospitalization for HF. SGLT-is significantly reduce the risk of HF by 30–40% in individuals with T2D with established or high risk of CVD according to previous large-scale RCTs, and the HF benefits exists no matter the ejection fraction is preserved or reduced.^297–299,301^ Subsequent trials in patients with T2D and established HF showed that dapagliflozin and empagliflozin reduced the composite outcomes of worsening HF (defined as hospitalization for HF or an urgent visit for HF) or cardiovascular death by 21–26%.^303–306^ SGLT-i sotagliflozin in the SOLOIST-WHF trial was associated with a 33% reduction in worsening of HF, which is greater than other SGLT-is.^307^

Most GLP-1 trials did not significantly reduce the risk of HF in patients with T2D.^286–292^ Despite the benefits of ASCVD, pioglitazone may increase the risk of HF. Many RCTs and meta-analyses have shown that TZDs, including pioglitazone, increase the risk of HF; however, it seems that an increased risk of HF does not increase the risk of death or cardiovascular events following HF.^308–312^ This may be explained by the fact that an increased risk of HF may be related to fluid retention, which suggests that this change is reversible. Moreover, there is no direct evidence that TZDs damage the structure and function of the heart. Instead, some animal experiments and clinical trials have suggested that TZDs might improve ventricular structure or function.^280,313,314^ SGLT-is have become the first choice of hypoglycemic agent for patients with T2D and HF or for those with multiple risk factors for HF due to its well-proven benefits.^211^

Meta-analysis and post hoc analysis demonstrated that the cardiovascular benefits of GLP-1 and SGLT-is are not associated with the use of metformin; therefore, in patients with established CVD or at high risk of CVD, metformin is not the basic therapy.^1,315,316^

Although there were no head-to-head RCTs comparing GLP-1 and SGLT-is for cardiovascular benefit, a meta-analysis showed no significant difference between them.^1^ Recently, a propensity pair-matched study showed that combination therapy comprising GLP-1 and SGLT-is reduced the risk of all-cause mortality and CVD better than did GLP-1 or SGLT-is alone.^317^ The mechanism of SGLT-is and GLP-1 on CVD benefits might be that they improve mitochondrial energy metabolism and reduces oxidative stress though activation of AMPK signaling pathways^318^; and alleviate inflammation through reduced proinflammatory factors, infiltration of immune cells and downregulated NF-κB and JAK/STAT signal pathways. In addition, the anti-inflammatory effects also gain benefits on skeletal muscle atrophy and retinal vascular permeability.^319–322^ SGLT-is can also reshape gut microbiota, reducing uremic toxins on organs such as heart and kidney^323^ and upregulating l-tryptophan which promote secretion of GLP-1.^324^ GLP-1 similarly reduce atherosclerosis by reducing inflammatory factors. In addition, GLP-1 can reduce cardiomyocyte apoptosis and increases left ventricular ejection fraction through AMPK activation and NF-κB downregulation.^318^

##### Hypoglycemic agents for patients with T2D and CKD

CKD, diagnosed by a persistent increase in urinary albumin excretion (albuminuria), a low estimated glomerular filtration rate (eGFR), or other manifestations of kidney damage, may be a consequence of DN and nondiabetic nephropathy coincident with T2D.^1,325^ CKD can progress to end-stage renal disease (ESRD), which requires dialysis or kidney transplantation and increases the risk of CVD. SGLT-is have been proven to be beneficial for CKD in many trials. Several trials of SGLT-is in which renal efficiency was set as a secondary outcome have shown that SGLT-is reduces the risk of worsening CKD.^297,298^ Additionally, two other key trials of SGLT-is in people with CKD showed that canagliflozin reduced the primary composite outcome of ESRD (dialysis, transplantation, or a sustained estimated eGFR <15 ml per minute per 1.73 m^2^), doubled the serum creatinine level, or led to death from renal or cardiovascular causes by 30% in the CREDENCE trial, and dapagliflozin reduced the risk of a composite outcome of a sustained decline in eGFR of at least 50%, ESRD, or death from renal or cardiovascular causes by 39% in the DAPA-CKD trial compared with the placebo. This might due to the ameliorated proximal tubular cells apoptosis through glycine-mediated activation of AMPK/mTOR signaling pathway.^326^Besides SGLT-is can also inhibit apoptosis in kidney through PI3K/Akt signaling pathways^327^ and renal tubular ferroptosis though AMPK/NRF2 signaling pathways.^328^

GLP-1 has also been demonstrated to have renal benefits.^329,330^ The LEADER, SUSTAIN 6 and REWIND trials, which examine cardiovascular outcomes, showed that liraglutide, semaglutide and dulagutide, respectively, reduced the risk of a composite renal outcome by 22%, 36%, and 15%, while ELIXA and EXSCEL trials demonstrated less than a 20% risk reduction, with no significant difference compared with that of the placebo.^287,289–292^ These results are limited to patients with CVD and cannot be generalized to people with CKD. The FLOW trial firstly demonstrates that semaglutide reduced the risk of major kidney disease events by 24% in the population with T2D and CKD.^331^ Another study reclassified FLOW trial by whether or not SGLT-2 was used, and the analysis found consistent renal benefits of semaglutide with or without SGLT-2i. But further study specifically targeted for verification of combined effect are needed, as the FLOW only has limited test power due to fewer people using SGLT-2i in this trial.^332^ The potential mechanism of renal benefits be reduced oxidative stress through decreased levels of glomerular superoxide and renal NADPH oxidase and reduced inflammation through counteracting angiotensin-II- induced NF-κB activation in glomerular endothelium and mesangial cells. In addition, GLP-1 induced human mesangial cells reduced proliferation and fibrosis.^333^ For now, SGLT-is are the only recommended first choice for patients with T2D and CKD or with multiple risk factors.^1^

##### Hypoglycemic agents for T2D patients at medium risk of CVD

For diabetic patients at medium risk of CVD, referring to patients without other CVD and CKD risk factors other than diabetes, the choice of hypoglycemic drugs emphasizes the effect of glycemia control and weight management. For preobese/obese patients, a class of hypoglycemic agents with weight loss effects are recommended, with dulaglutide as the first-line therapy. For nonobese or nonobese patients with T2D, if there is no concern about weight loss, the abovementioned recommendation also applies to this population. In patients with weight loss concerns, classes of hypoglycemic agents with neutral effects on body weight or weight gain are recommended, such as DPP-4i, sulfonylureas, and glinides.^211^

##### Hypoglycemic agents for T2D patients at high hypoglycemic risk

Hypoglycemia is a rare but serious consequence of T2D, and poor glycemic control, glycemic variability, kidney damage and poor cognitive function are strongly associated with an increased risk of hypoglycemia.^334^ Patients with newly diagnosed T2D without CKD are usually not at high risk of hypoglycemia. T2D patients who are elderly and have renal failure or severely impaired liver function are common high-risk groups for hypoglycemia; therefore, the hypoglycemic risk of drugs should be considered when choosing medications. For T2D patients with renal function or severe liver function impairment will be discussed in a later part. For elderly individuals who are preobese/obese or have a weight loss demand, metformin, AGI, SGLT-is and GLP-1 (liraglutide, dulaglutide, and semaglutide) are recommended because of their low hypoglycemic risk and weight loss benefits. For elderly patients without preobesity/obesity and with weight loss concerns, the use of DPP-4i is recommended because of its neutral effect on body weight.^211^

##### Hypoglycemic agents for T2D patients with hepatic failure

Diabetic patients with severely impaired liver function are prone to fasting hypoglycemia due to impaired liver gluconeogenesis. In patients with severely impaired liver function, the choice of hypoglycemic agents should emphasize the low risk of hypoglycemia and decreased liver impairment associated with hypoglycemic drugs. Therefore, when patients have severe liver function impairment, insulin, linagliptin, lixisenatide, and dulaglutide are recommended.^211^

##### Hypoglycemic agents for T2D patients with renal failure

People with diabetes and CKD are at high risk for progression to kidney failure and cardiovascular events, as well as acute hypoglycemia and diabetic ketoacidosis (DKA). The hypoglycemic agents with renal benefits have been described in the section “Hypoglycemic agents for patients with T2D and CKD”. For T2D patients with a moderate or greater renal function decrease (eGFR <45 ml/min/1.73 m^2^), hypoglycemic agents should be carefully selected. When renal failure reaches the G3b stage (eGFR 15–29 ml/min/1.73 m^2^), DPP-4i and sulfonylureas (gliclazide and glipizide) need to be administered at lower doses, while glibenclamide and glimepiride are not allowed. When renal failure reaches the G4 stage (eGFR 15–29 ml/min/1.73 m^2^), metformin, exenatide, lixisenatide, sulfonylureas and AGIs are not recommended, and these medications should be discontinued or not initiated. When renal failure reaches the G5 stage (eGFR <15 ml/min/1.73 m^2^), which requires dialysis or transplantation, GLP-1 and TZD should be discontinued, while the DPP-4i linagliptin and insulin might be the preferred choices.^335^

SGLT-is can be initiated in diabetic patients with renal failure at any stage but for different treatment purposes. An eGFR >45 ml/min/1.73m^2^ and a urinary-to-creatinine ratio (uACR) ≥25 mg/mmol or 20–45 ml/min/1.73 m^2^ or established CVD are recommended for diabetic patients because of its high glucose-lowering efficacy and proven cardiorenal benefits. In addition, SGLT-2i is also suggested for patients with an eGFR >45–60 ml/min/m^2^ and a uACR <25 mg/mmol to improve cardiovascular risk and slow the rate of renal function decline but have limited effects on glycemic control. For eGFRs <20 ml/min/m^2^, there are some inconsistencies. The guidelines issued by the Kidney Disease Improving Global Outcomes (KDIGO) suggest that SGLT-2i should be discontinued, while the 2023 guidelines issued by the UK Kidney Association (UKKA) suggest initiating SGLT-2i to slow the progression of kidney disease.^335,336^

##### Initial insulin therapy

As the disease progresses, patients with T2D eventually develop an absolute lack of insulin and require insulin therapy. For newly diagnosed T2D patients, insulin therapy should be considered when HbA1c >10% is associated with symptoms of hyperglycemia or ketonemia, as indicated in the treatment stratification above. Basal insulin alone is the most convenient initial insulin therapy, but it can also be used in combination with other drugs for glycemic control, which allows for both effective glycemic control and additional benefits according to the patient’s characteristics.^1^ When diabetic patients are not at risk of DKA, basal insulin plus noninsulin hypoglycemic agents with weight loss effects are recommended for those with preobesity/obesity or without weight loss, and basal insulin/premix insulin plus secretagogues or DPP-4i are recommended for those without preobesity/obesity and with weight loss concerns. When diabetic patients are at risk of DKA, multiple daily injections of insulin are more appropriate. Once DKA develops, emergency actions should be taken immediately, which will not be discussed here.^211^

##### Combination therapy

**Fig. 4:**
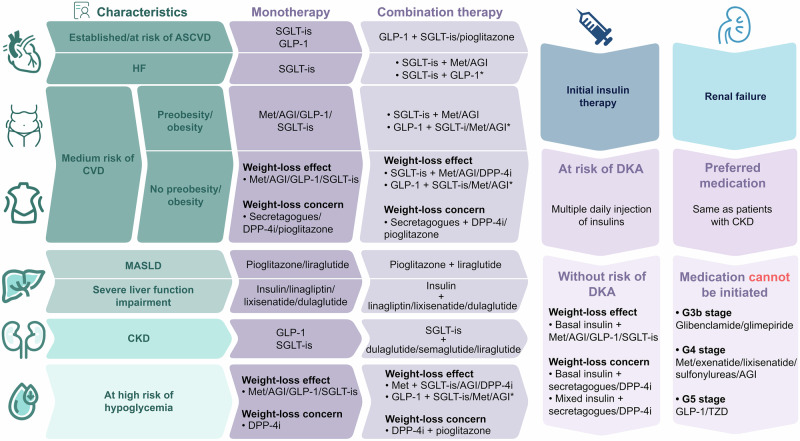
Personalized choice of hypoglycemic agents for patients with newly diagnosed T2D. This figure is modified according to Zhang et al.^211^ T2D type 2 diabetes, MASLD metabolic associated steatotic liver disease, ASCVD atherosclerotic coronary heart disease, HF heart failure, CKD chronic kidney disease, CVD cardiovascular disease, DKA diabetic ketoacidosis, Met metformin, AGI α-glucosidase inhibitors, GLP-1 glucagon-like peptide-1, SGLT-is sodium-glucose cotransporter inhibitors, DPP-4i dipeptidyl peptidase-4 inhibitors, TZD thiazolidinediones. *These regimens are considered in patients with affordability and willingness for injection therapy

For people newly diagnosed with T2D, there are two approaches to pharmacal therapy: stepwise therapy and combination therapy. Stepwise therapy starts with monotherapy followed by stepwise addition of add-on medication to the main glycemic targets; this approach is a conventional therapy and has been well studied.^337^ The choice of hypoglycemic agents for initial monotherapy should be personalized considering the need to reduce MDS-related TOD, such as weight management and comorbidities, as demonstrated above. Initial combination therapy is considered for those with a present HbA1c level > target HbA1c + 1.5% but ≤10%, and emerging evidence has shown that combination therapy might offer some potential benefits. Multiple trials have demonstrated that combination therapy with complementary mechanisms reduces HbA1c more quickly and increases with faster achievement of glycemic targets.^338–340^ Results have shown that glucose-dependent organ protection agents (saxagliptin combined with metformin) have beneficial effects only on islet β cells but no protections of target organs has been showed, as the VERIFY^341^ trial showed that combination therapy comprising DPP-4i and metformin reduced the risk of treatment failure (defined as HbA1c > 7% at two consecutive scheduled visits 13 weeks after randomization) by 49% compared with that of stepwise therapy, and it extended the duration of glycemic maintenance by 2 years, which suggested that the initial combination therapy delayed the deterioration of islet β cells. A real-world prospective cohort study including 2.2 million people demonstrated that the combination of SGLT-i and GLP-1 had better effects on all-cause mortality, MI and admission rate than SGLT-i or GLP-1 alone.^317^ Combination therapy should also be personalized according to patient characteristics as monotherapy does. Fig. 4 shows the combinations of different hypoglycemic agents.

We summarized the personalized selection of hypoglycemic agents for patients with newly diagnosed T2D according to different clinical scenarios in Fig. 4.

### Treatment of MDS-related TOD/complications in patient with T2D

#### MDS-related chronic kidney disease

We believe that, unlike classical T1D, T2D is a component of MDS and chronic kidney disease in T2D patients is supposed to be TOD/complications of MDS, since any component of MDS can cause the initiation and progression of CKD.^342^ Moreover, a considerable number of patients with T2D also have hyperuricemia, which is also a cause of CKD.^343^ Therefore, it is not appropriate to use “diabetic kidney disease” in this case and it may be more appropriate to use MDS-related CKD (MDSRCKD) for most T2D patients after excluding other specific causes. The use of this term is conducive to its prevention and control for early intervention with every risk factor of MDSRCKD instead of the conventional glycemic-control-centered management. In addition, the presence of CKD increased cardiovascular risk and mortality in patients with diabetes.^1,344^

##### Holistic management

Both the uACR and eGFR are two common and easily performed markers for early diagnosis and monitoring of CKD progression and should be assessed annually for all patients with T2D, and 1–4 times per year for patients with established CKD to monitor the development of renal function.^1^

*Nutrition*. For patients requiring no dialysis, restricting protein intake to 0.8 g/kg per day is appropriate since this level slows the reduction in eGFR while higher or lower level showed no better or even worse effect. For patients requiring dialysis, protein intake should be elevated to 1.0-1.2 g/kg per day since they may suffer from malnutrition. Control of dietary potassium and sodium is necessary for the balance of serum potassium and sodium, since patients with reduced eGFR may have a problem with urinary excretion of potassium and sodium. Additionally, dietary potassium and sodium should be controlled according to the comorbidities, medicine use, blood pressure and test results.^1^

*Glycemic control*. Evidence from various studies have proved that glucose-lowering can delay the onset and progression of CKD. Moreover, there have been hypoglycemic agents such as SGLT-is have been proven to have direct renal benefits independent of glucose. Non-pharmacological therapies and pharmacologic therapies for patients with CKD have been fully discussed in parts above.

We believe it is unreasonable to recommend metformin for diabetic patients with CKD as we have not found evidence of additional renal benefits from metformin for diabetic patients with CKD. In addition, for those with an eGFR <60 ml/min/1.73 m^2^, glycemic target should be relaxed due to the increased risk of hypoglycemia.

*Antihypertension therapy*. Hypertension is a strong risk factor for the development and progression of CKD. Antihypertension therapies have been proven to slow the progression of CKD in patients with diabetes and reduce the risk of cardiovascular events, and a blood pressure <130/80 mmHg is recommended to achieve such benefits. In patients with diabetes and hypertension, angiotensin-converting enzyme (ACE) inhibitors or angiotensin receptor blockers (ARBs) are the preferred first-line agents for treating hypertension for those with an eGFR <60 ml/min/1.73 m^2^ and a uACR ≥300 mg/g creatinine for their proven effect to slow the progression of CKD and reduce the risk of cardiovascular events.^1^ Moreover, other antihypertension agents lacking evidence of renalcardiovascular benefits could be added for personal blood pressure targets.

*Mineralocorticoid receptor antagonists (MRAs)*. MRAs have not been well studied in CKD before due to their risk of hyperkalemia. Currently, the novel nonsteroidal MRA finerenone has been demonstrated to delay the progression of CKD and reduce cardiovascular events in patients with T2D and CKD in FIDELIO-DKD trial and FIGARO-DKD trials which primarily focus on renal outcome and cardiovascular outcome, respectively. These two trials demonstrated that compared with placebo, finerenone reduced the worsening of CKD by 23%, and also reduced the risk of cardiovascular events by 14%. However, compared with placebo, finerenone also showed a greater risk of hyperkalemia.^345,346^ In patients with T2D and CKD, finerenone, the only nonsteroidal MRAs with proven renalcardiovascular benefits, was considered for those with an ACR ≥ 30 mg/g and normal potassium.^1^

*Control of other risk factors*. Dyslipidemia is associated with the pathogenesis and progression of CKD. Moreover, as renal function declines, the lipid profile gradually shifts to uremic lipid profile, which is characterized by increased triglycerides, low HDL cholesterol and elevated LDL cholesterol and contributes to atherosclerosis.^347^ Therefore, lipid-loss management is essential for potentially delaying the progression of CKD and reducing the cardiovascular risk. Moderate- or high-intensity statin is recommended for all patients with T2D and CKD. Statin therapy is used for secondary prevention of CVD and for primary prevention in individuals aged >40 with diabetes or with CKD stages 1–4 and kidney transplantation. In addition, aspirin should generally be used for patients with established ASCVD and considered for primary use for those at high risk of ASCVD.^335^

#### DN

DN comprises a heterogeneous group of disorders that affect different parts of the nervous system and present with diverse clinical manifestations.^1^ DN is believed to be associated with neuronal, neuroglial cell and vascular endothelial cell injury caused by uncontrolled hyperglycemia, dyslipidemia and IR through multiple pathways, such as ERS, inflammation, oxidative stress and mitochondrial disorders.^70^ DPN and diabetic autonomic neuropathy, especially cardiovascular neuropathy (CAN) are the most common and well-studied forms of neuropathy.

Screening at the time of diagnosis and annually thereafter is important for detecting DN in a timely manner and preventing further damage to the nervous system. Current treatments are not able to reverse the existing nerve damage but rather delay the onset and progression of DN and relieve symptoms to improve life quality.

##### Pathology-targeting treatment

*Glycemic control*. Several lines of evidence have shown that intensive glycemic control dramatically reduced the risk of DPN and CAN in patients with T1D; however, some studies have demonstrated limited benefits of intensive glycemic control for T2D patients as only modestly prevents or delays the progression of DPN and CAN in patients with T2D, possibly because of undiagnosed hyperglycemia and multiple combined risk factors.^1^

*Lipid management*. Emerging evidence has demonstrated that dyslipidemia plays a crucial role in the pathogenesis of DN.^70^ Various strategies for lowering lipids, such as physical activity, weight loss and bariatric surgery, have been reported to have positive effects, but the use of lipid-lowering medicine does not seem to be effective at slowing the development of DN.^348^

*Hypertension control*. Many studies have shown that hypertension is also involved in the development of DN. A meta-analysis revealed that hypertension is an independent risk factor for DPN. Additionally, data from ACCORD trial demonstrated that intensive blood pressure management reduced the risk of CAN by 25%.^1^

##### Symptom-alleviating treatment

*Neuropathic pain*. Symptoms of DPN vary according to the class of involved sensory fibers, presenting with pain and unpleasant sensations if small fibers are involved and with numbness and loss of protective sensation (LOPS) if large fibers are involved.^1^ Severe neuropathic pain can significantly limit motivation, and impact life quality and contribute to depression and social dysfunction. However, there is insufficient evidence indicating that holistic management of glycemia and other risk factors can alleviate the neuropathic pain. Therefore, agents for treating neuropathic pain are essential to relieving neuropathic pain, improving life quality and mental health. A number of agents can be considered for pain in DPN patients. Gabapentinoids, serotonin-norepinephrine reuptake inhibitors (SNRIs), sodium channel blockers, and tricyclic antidepressants have been suggested as the first approach for neuropathic pain in patients with DPN, and oral analgesics and lidocaine patches are also considered.^1^

*Diabetic autonomic neuropathy*. Diabetic autonomic neuropathy affects autonomic neurons, and the symptoms vary according to the organs or systems of autonomic neuron damage. As with DPN, management targeting pathogenesis is not able to reverse established autonomic neuropathy. Treatment for existing diabetic autonomic neuropathy generally focuses on alleviating the symptoms. For example, CAN often presents with resting tachycardia and orthostatic hypotension, so nonpharmacologic strategies (such as adequate salt intake and physical activities and avoidance of medicine-related hypotension) and pharmacologic strategies (such as midodrine and droxidopa) should be considered to minimize postural symptoms rather than to restore normotension.^1^ When it affects the gastrointestinal system, patients can exhibit esophageal dysmotility, gastroparesis, constipation, diarrhea, and fecal incontinence.^349^ Dietary modifications and pharmacologic strategies targeting symptoms are considered to improve life quality despite no alterations in the underlying pathology or natural history of this disease.^1^

#### DFU

DFU is the advanced consequence of multiple factors, including uncontrolled hyperglycemia, DPN and peripheral arterial disease (PAD). It is the leading cause of disability and mortality in people with diabetes.^1,350^

##### Prevention

Preventing the development of foot ulcerations for the first time and after healing plays an essential role in the management of foot ulcerations. Early recognition of risk factors and examinations for foot ulcerations are effective ways to prevent and delay the occurrence of DFU and amputation. DFU are associated with multiple factors including DPN, PAD, foot deformities, history of foot ulcerations or amputations, nephropathy, etc., and are stratified at risk based on these risk factors. A thorough examination of the feet including the evaluation of DPN, PAD, and foot deformities should be performed annually in all people with diabetes, and the interval should be shortened based on the level of risk.^1^

*Education*. For all diabetic patients, especially those at risk of foot ulcerations, education on foot care, including disease awareness and self-care strategies, should be provided. However, a systematic review demonstrated that education about foot care improved the awareness and acknowledgement of foot ulcerations and the care, but it failed to achieve a positive impact on self-care behavior and incidence of diabetic foot ulcerations or amputation.^351^

*Foot care*. Foot care for patients with diabetes should be determined by their risk category. Diabetic patients at no or low risk can be managed by education and self-care. Diabetic patients at moderate or high risk require the involvement of a foot care specialist for further examination and surveillance. Treatment should include daily foot inspection, the use of moisturizers, the avoidance of self-care of ingrown nails and calluses, and specialized shoes (such as shoes with customized pressure-relieving orthoses for people with increased plantar pressures; additionally, individuals with deformities such as bunions or hammertoes may require specialized or custom-made footwear for people with deformities). Those who with neuropathy present with a warm, swollen, red foot requires a thorough work up for possible charcot neuroarthropathy, nonweight bearing and urgent referral to a foot care specialist to prevent foot deformities and instability leading to ulcerations and amputations.^1^

##### Treatment of foot ulcerations

There are five basic principles for treating foot ulcerations which includes offloading of plantar ulcerations, debridement of necrotic, nonviable tissue, revascularization of ischemic wounds when necessary, management of infection, and use of physiologic, topical dressings.^1^ Effective off-loading prevents patients with plantar neuropathic ulcers from walking on the lesions and is the key to successful management.^349^ If the ulcers fail to show a ≥50% reduction after 4 weeks of appropriate management via conventional methods, several advanced therapies, including negative-pressure therapy, growth factor therapy, bioengineered tissue, acellular matrix tissue, stem cell therapy, hyperbaric oxygen therapy, and, most recently, topical oxygen therapy, can be considered. However, these robust RCTs for these advanced therapies are lacking, which is challenging. Once the wounds healed, the above prevention program was started.^1^

#### DR

DR is strongly related to the duration of diabetes, the level of glycemic control, nephropathy, hypertension and dyslipidemia and contributes to the disruption of the blood-retinal barrier (BRB), which is mainly caused by VEGF-A and proinflammatory factors.^1^ DR remains to be the leading cause of visual loss and blindness globally.^352^

##### Holistic treatment

It is essential for patients to be screened for DR once diagnosed with T2D and undergo regular eye examination by an ophthalmologist since early diagnose and treatment can efficiently slow the progression of DR and prevent and prevent vision loss. The time interval of examination depends on the presence of risk factors for the onset and worsening of DR such as uncontrolled hyperglycemia and diabetic macular edema.^1^

Education for awareness and health care and control of hyperglycemia, dyslipidemia and hypertension are key strategies for slowing the onset and worsening of DR, similar to other MDS-related complications.

##### Ophthalmic treatment

Ophthalmic treatment requires an ophthalmologist with experience in DR treatment. There are two main treatment strategies: photocoagulation surgery and intravitreal drug injection.

*Photocoagulation surgery*. Panretinal laser photocoagulation therapy is a common strategy for treating high-risk PDR and some severe nonproliferative diabetic retinopathy (NPDR) since evidence shows that it reduces the risk of severe vision loss from PDR in patients with high-risk PDR and some severe NPDR. The macular laser photocoagulation technique, which is more gentle, was shown to be effective in treating diabetic macular edema.^1^

*Intravitreal drug injections*. VEGF-A is upregulated due to MDS and partially contributes to the vascular changes in DR by altering the blood vessel permeability and promoting neovascularization.^352^ Intravitreal injection of anti-VEGF agents has provided an effective alternative to panretinal laser photocoagulation therapy for PDR. Evidence has demonstrated that the intravitreal injection of anti-VEGF agents effectively reduces the risk of vision loss. In addition, it is more effective to treat center-involved diabetic macular edema than photocoagulation surgery and has become the first-line therapy for diabetic macular edema.^1^ Notably, not all patients respond optimally, indicating that other mechanisms are involved in vascular changes in DR.^352^

A variety of studies suggest that proinflammatory factors such as IL-1β, TNF α, and IL-6 may contribute to DR pathogenesis.^352^ Therefore, intravitreal injection of corticosteroid is an option for patients who are not candidates for anti-VEGF therapy.^1^

## Conclusion and perspective

It is hoped that this review and our idea can provide a reference for most newly diagnosed patients with T2D receiving hypoglycemic therapy. The mechanism of T2D is not fully understood, and there is no cure yet. However, as mentioned earlier, T2D and MDS-related TOD are preventable, and effective measures must be taken to prevent the occurrence of diabetes, such as the prevention and treatment of preobesity and obesity and MASLD, which will greatly reduce the occurrence of T2D. If diabetes prevention fails, early detection should be emphasized. Once diabetes is diagnosed, holistic management of organ protection targeting MDS should be provided since most patients with T2D also have MDS. Due to the article layout limitation, we did not specifically address the prevention and treatment of hyperglycemic crises, DKA or the hyperosmolar hyperglycemic state (HHS). Diabetes treatment available for elderly individuals or those with gestational diabetes was not covered in this review. We also did not discuss much about the management of patients with T2D and CVD. We believe that T2D as a disease is simple, but the situation of patients with T2D is more complicated since most of them have MDS, and T2D is only one of the components that usually occurs later than preobesity, obesity and MASLD. Therefore, the term “complications” should not be used arbitrarily in patients with T2D, especially for “DKD” “macrovascular complications”; otherwise, the management of MDS will be mistakenly guided to focus on glycemic management. Only by holistically managing MDS can we achieve our goals of improving the quality of life and prolonging the life expectancy of patients with T2D.
